# Perceptual and Gaze Biases during Face Processing: Related or Not?

**DOI:** 10.1371/journal.pone.0085746

**Published:** 2014-01-15

**Authors:** Hélène Samson, Nicole Fiori-Duharcourt, Karine Doré-Mazars, Christelle Lemoine, Dorine Vergilino-Perez

**Affiliations:** 1 Laboratoire Vision Action Cognition (Institut de Psychologie, Institut Universitaire Paris Descartes de Psychologie & INC), Université Paris Descartes & Sorbonne Paris Cité, Boulogne Billancourt, France; 2 Institut Universitaire de France, Paris, France; University of Leuven, Belgium

## Abstract

Previous studies have demonstrated a left perceptual bias while looking at faces, due to the fact that observers mainly use information from the left side of a face (from the observer's point of view) to perform a judgment task. Such a bias is consistent with the right hemisphere dominance for face processing and has sometimes been linked to a left gaze bias, i.e. more and/or longer fixations on the left side of the face. Here, we recorded eye-movements, in two different experiments during a gender judgment task, using normal and chimeric faces which were presented above, below, right or left to the central fixation point or on it (central position). Participants performed the judgment task by remaining fixated on the fixation point or after executing several saccades (up to three). A left perceptual bias was not systematically found as it depended on the number of allowed saccades and face position. Moreover, the gaze bias clearly depended on the face position as the initial fixation was guided by face position and landed on the closest half-face, toward the center of gravity of the face. The analysis of the subsequent fixations revealed that observers move their eyes from one side to the other. More importantly, no apparent link between gaze and perceptual biases was found here. This implies that we do not look necessarily toward the side of the face that we use to make a gender judgment task. Despite the fact that these results may be limited by the absence of perceptual and gaze biases in some conditions, we emphasized the inter-individual differences observed in terms of perceptual bias, hinting at the importance of performing individual analysis and drawing attention to the influence of the method used to study this bias.

## Introduction

Faces are of crucial interest as they create and fashion our social interactions. By looking at faces, we are able to extract much information very quickly such as gender, age, emotional state, identity and even personality traits of individuals [1], [2]. Since the advent of the first model for face processing presented by Bruce and Young [3], many researchers have investigated the way people process facial identities, expressions or features [4]–[7] and their cerebral bases [8]–[10]. Lately, the recording of eye movements was used to observe the early stages of face processing (i.e., scanning while encoding a face) and the cognitive strategies that may be expressed in the scanning pattern. As looking at faces is an overlearned skill acquired from birth, observers tend to develop similar patterns of exploration focusing on particular features depending on the stimulus-driven saliency and/or the task's demands (e.g. [11]; see also citations in [12]). For example, where a face identification task involves a pattern of fixations distributed all over the face, a gender judgment involves longer fixations on the face's eyes (unpublished data cited in [13]). In this study, we focus on the relationship between the perceptual and oculomotor processes that are involved in face perception, using a facial gender judgment task.

Most studies looking at gaze strategies during face exploration have reported a bias for looking toward the left side of the face (Here, we will consider and speak from the observer's point of view: i.e. a left bias means that the observer looks at or responds considering the left side of the face, i.e. the right half-face (from the observer's point of view).) (e.g. [14]–[17]), although others did not find it (see [[18], [19]] described later). Mertens et al. [20] proposed that this bias might be due to an internal factor because it was only observed with faces but not with any other symmetrical objects such as vases. Similarly, Leonards & Scott-Samuel [12] reported a general leftward bias for the first saccade directed to the face that was not found in the exploration of landscapes or fractals. If those previous studies used central presentation of faces, it was shown only recently that when subjects were requested to make an initial saccade toward a face presented either on the top or the bottom of the screen, the preferred landing position on the face was again slightly set to the left of its center [21]. Such consistent left gaze bias (GB) has been observed irrespective of the task demands –judgment of expression, familiarity or free viewing [22], [23], or regarding the observed faces - humans or animals [23]. Indeed, Guo et al. [24] have recorded eye movements from 6-month-old infants to adults, rhesus monkeys and domestic dogs while viewing neutral human face images, monkey faces and inanimate objects. All faces were presented upright and upside-down. Gaze asymmetry was observed in humans and in animals. The left side of the face was not only the preferred landing area for the first fixation position, but it had a higher proportion of viewing time for all facial stimuli. However, human infants showed a general leftward bias for all upright images, while adults showed a specific bias toward the left side of the face for upright human faces only, suggesting a development of the left GB over time.

The left GB may reflect a right hemisphere bias for face processing, as shown by studies based on brain imaging methods in patients with focal brain lesions, normal subjects [10], [25], [26] or by the impairment of face recognition in prosopagnosic patients after right or bilateral acquired brain injury [27], [28]. The right hemisphere dominance for face processing also appears in perceptual tasks where participants must assess the age, expression, attractiveness, identity or gender of chimeric faces, i.e. faces composed of different left and right hemi-faces. In such tasks, many studies have highlighted a left perceptual bias (PB), as observers tend to ground their responses on the left side of the face [14], [29]–[33]. Even though the left GB was found to be consistent regardless of the task, the magnitude of the left PB was dependent on the task as well as on the complexity of the stimulus. For example, Burt & Perrett [29] have reported that 77% of the responses were based on the left side of faces in an age judgment task, against 67% for a gender judgment and 58% for an expression judgment task. In a similar way, Coolican et al. [30] have found a greater left PB in an emotion judgment task than in an identity judgment task. Furthermore, in an emotion judgment task using chimeric faces of varied complexity – easy, medium, difficult- relative to the accentuation of the emotion carried by the face, Carbary et al. [34], [35] observed that in adults, the left PB was reduced when the task was judged to be difficult due to the face complexity. The authors accounted for this result in terms of hemispheric specialization: a configural processing subtended by the right hemisphere for the easiest task and a featural processing for the most difficult task. Another argument in favor of a RH implication for the left PB is the finding of Coolican et al. [30] regarding perceptual judgments of emotion with upright and inverted chimeric faces. They observed a reduction or even a lack of left PB for inverted faces, which suggests that configural processing underlies the left PB. Finally, several studies in infant and adult patients, with right or left hemispheric lesions, have shown different patterns in processing chimeric faces. Young infants with early focal brain lesions of the right hemisphere (RH) showed a left PB which depended on the severity of their lesion: a reduced bias when the lesion was moderate and no left PB but a right one when the lesion was the most severe. For children with left hemisphere (LH) damage, the left PB was preserved, even though it was slightly reduced with regard to the control group [36]. Adults with late acquired lesions of the LH show stronger left PB while adults with RH damage show a right PB [30], [37]. A more direct relation between left PB and RH dominance for face processing was given recently by Yovel et al. [38]. They showed correlations between the asymmetry of the volume of the fusiform face area (FFA), recorded in a fMRI session during the viewing of chimeric faces, and the magnitude of the left PB measured outside the scanner with an identity judgment task. Nine out of seventeen participants showing a left PB had a larger face-selective activation over the right FFA compared with the left FFA whereas two subjects out of six showing a right PB had a larger contralateral left FFA compared with right FFA. Finally, there was no difference between right and left FFA activations in one subject showing no PB.

All together, these studies lean towards a RH dominance for facial processing to account for the left PB or GB. However, these results do not show that both left PB and GB are closely linked. Indeed, it may be expected that the left PB may be due to the left GB: the perceptual decision may be taken on the left side of the face because fixations are more frequent and longer on this side. Alternatively, the inverse relationship may exist: the gaze is directed to the left side of the face because it represents the more “salient” part of the face. Arguments in favor of this second possibility come from studies using “bubbles technique” in face recognition or gender identification tasks suggesting that local facial features on the left side of the face (e.g. the left eye) are the earliest diagnostic feature used by the participants to perform the tasks [39], [40]. Finally, it is also possible that no direct link exists between both perceptual and gaze biases, both being due independently to a more central RH bias in visuo-spatial processing. Only few studies have specifically examined the link between left perceptual and gaze biases. This was done by Grega et al. [41] in a task requiring a similarity judgement between whole faces and composite chimeric stimuli. Although they found a left PB, they failed to find any consistent GB on the first saccade or on the gaze duration. In contrast, Phillips & David [17], who examined the link between the left PB and the left GB in normal control participants and schizophrenic patients performing a recognition task, did not find any PB but noted a significant left GB for control participants across stimuli but only a non-significant trend across subjects. Conversely, schizophrenic patients viewed the right side of faces first and did not present any PB. Butler et al. [14] recorded the eye movements of participants who freely explored a central chimeric face for two seconds to judge of its gender. Overall, they observed a left PB in their experiment as well as a left GB on the initial saccade as 75% of the initial saccades were directed to the left side of the face. They explained this result as a tendency to explore, at first, the side of the face better suited for face analysis (the left visual hemi-field projecting to the RH). However, they did not observe an overall left GB when they analyzed the fixations durations and only a marginal left GB on the number of total left/right fixations was present. When they examined separately the GB for left and right PB, they still failed to find any strong relation between PB and GB on the first saccade: 77% of the first fixations for left PB and 71% for right PB were directed to the left. However, they recorded more subsequent left saccades and longer fixation durations on the left for left PB whereas the number of saccades to the left or to the right side of the face and the fixation duration did not differ for trials with a right PB. Butler & Harvey [42] further explored this link and reported a left PB even when the face was displayed too briefly to allow any eye movements (100 ms). However, they noted that the left PB observed during this short presentation time was less pronounced than the left PB noted during the saccadic condition (viewing faces for 2 seconds, [14]) as respectively 55% against 63% of the responses in the gender task were based on the left side of the face. In a third study looking at the evolution of the PB with the increase of face exposure duration (100 ms, 300 ms or free viewing) in young and old adults [43], they found again an increase of the magnitude of the left PB with the exposure duration (for young adults, 56% of the responses were based on the left side of the face in the 100 ms condition, 59% in the 300 ms and 58% in free viewing). They concluded that eye movements are not required to generate the PB although they help to reinforce it: when the face is presented centrally with an initial fixation on its center, the left side of the face is projected to the RH that responds preferentially to face stimuli, involving the initial left PB. Then, the oculomotor system drives the gaze to the most salient side of the face as it can be shown by the GB on the first saccade as well as on the subsequent ones.

Although convincing, the results of Butler and collaborators raise some questions. In the light of recent results, one may ask whether the left GB found on the initial saccade is the sign of the RH dominance on the face processing or more simply linked to well-known effects on the saccadic control such as the optimal viewing position or the center of gravity effect. Indeed, Hsiao & Liu [44] recently showed that, similarly to the word recognition, the optimal viewing position for face recognition was slightly to the left of its center. Peterson & Eckstein ([45]) found similar results: the first saccade directed to a face landed to a location just below the eyes that maximized the perceptual performance in identity, gender or emotional judgment tasks. Such a location that slightly differed across perceptual tasks differed more substantially across observers, suggesting an “observer-specific synergy between the face-recognition and eye movement systems that optimizes face-identification performance” ([46]). Bindemann et al. [11] observed that when faces were displayed in many different views (frontal, mid-profiles and profiles), the observers fixated predominantly the eye and nose regions for frontal views, while for other views they fixated the innermost eye (mid-profile) or the only visible eye (profile). The first fixation always landed near the center of gravity of the face, and features were then targeted directly. Consequently the way observers look at faces depends on its orientation. Also, the left GB is not systematic and may change according to the method used in studies. This finding was also reported by Arizpe et al. [18] as they tested the effect of manipulating the starting position within a face on the subsequent fixations pattern during a face recognition task. In their work, the authors looked at the oculomotor exploration of centrally presented faces by manipulating the initial starting position (center of the face, right, left, top or bottom of the face corresponding respectively to the center of the nose, to the right earlobe, to the left earlobe, to the front or just below the chin). They found a high dependence of the fixation pattern on the start position. In particular, they showed that the first fixation landed near the center of the face with a slight tendency toward the starting position whereas the subsequent fixations landed on the facial side opposite to it. Thus, the tendency to saccade toward the left eye over right eye was reversed for a starting position on the left of the face. They explained their results on the initial saccade landing position by the center-of-gravity effect combined with the tendency for saccades to undershoot far targets or overshoot near targets (known as the “range effect”, [47], [48], [49]). Moreover, they showed that the central starting position commonly used in previous studies leads to first saccades with longer latencies compared to peripheral starting positions. This suggests that a greater amount of information is sampled before the first saccade, which could enhance the left biases introduced by the use of a central starting position on the face. This is consistent with recent results of Saether et al. [19] that failed to find any marked GB in a gender judgment task implying normal faces displayed in four different viewing angles in parafovea as the gaze always focused within an “infraorbital region” of the face whatever the viewing angle. They highlight that it is possible that “individuals largely differ in their perceptual biases in facial inspection and consequently that a specific directional bias might not be a particularly robust effect”. Indeed, as noted by some authors, although the left perceptual and gaze biases seem to be reliable phenomena at the group level, the analyses at the individual level reveal that not all subjects present such left biases. For example, Leonards & Scott-Samuel [12] noticed that their subjects had clear preference for one or the other hemi-field for the direction of their initial saccade since 60% of their subjects showed a left bias and 40% a right bias. Butler & Harvey [42] found a left PB for 13 out of their 17 subjects. Finally, Yovel et al. [38] also found consistent individual left, right and even no PB that correlated with the asymmetric activation of the FFA.

Taken together, these results indicate some heterogeneity. Two of the three studies looking at the relation between PB and GB failed to find either the PB or the GB [41],[17] whereas the third [14] found a subtle link between the two biases. The aim of our study is to further examine the PB and the GB and their possible relationship by manipulating two factors that seem to influence the PB and the GB: the face position and the number of saccadic eye movements that are required to explore the face. Moreover, we look more closely at individual PB and GB by reporting the number of participants presenting left/right/no biases.

## Experiment 1

In most experiments investigating PB or GB, the initial eye fixation was already within the face, as faces were centrally displayed (e.g. [14], [42], [43]). Recently, the results of Arizpe et al. [18] as well as Saether et al. [19] suggest that constraints regarding the experimental procedures using central face presentation may enhance the commonly reported biases. However, two studies still found a left perceptual bias for chimeric faces presented at the top and bottom of the central vertical axis ([50]) or in a binder [30] but none had recorded eye movements. Alternatively, by proposing top and bottom face positions forcing the first saccade to be driven by the face, Hsiao & Cottrell [21] observed again a first saccade landing slightly to the left of the nose. However, their experiment was not designed to look at the PB. This was also the case of Petersons & Eckstein ([45], [46]) that used peripheral starting locations to examine the first saccade landing position on a centrally presented face, in order to avoid possible bias due to the initial eye fixation within the face.

Then, in our first experiment, participants had to achieve a gender judgment task on normal and chimeric faces presented left, right, top or bottom to the central fixation cross, by remaining fixated to the screen's center throughout the trial or executing only one saccade to the face, a mask being displayed during the second saccade. Our interest in the fixation condition was to see whether the perceptual bias previously found with the central position may be modulated with other positions of face presentation: top and bottom positions in which each hemi-face was still presented in the left or the right visual field of the observer and left and right positions, for which, both hemi-faces were presented in the right or the left visual field respectively. Whereas we expected a left perceptual bias for top and bottom positions, we expected biases of proximity for left and right positions (i.e. left bias for right presentation and right bias for left presentation). In the saccade condition, we expected a modulation of the PB found in the fixation condition. This was based on the previous work of Butler and collaborators [43] showing an increase of the PB with the capacity to sample the faces. We also examined whether there was any link with the landing position on the face.

We used normal faces to ensure that participants would be able to distinguish a female from a male face, but also to control that our chimeric faces were processed in the same way as normal faces.

### Methods

#### Ethic Statement

The procedure was approved by the ethics committee of Paris Descartes University (Comité d'Evaluation Ethique en Recherche Biomédicale, CEERB, n°IRB 20130500001072).

#### Participants

Thirty-two young students (16 males and 16 females, *M* = 21.22±2.3 years) from Paris Descartes University were recruited for this study on a voluntary basis or for course credit. All of them signed a consent form, were Western European and right-handed (*M* = 92.21±6.2%), according to the Humphrey Laterality Questionnaire (modified by Hécaen & Ajuriaguerra [45]) and had normal or corrected to normal vision. They were subjected to the Mini Mental State Examination (score≥24) and the Beck Depression Inventory (score≤11) in order to filter out subjects with low cognitive level and mood disorders. The study complies with the principles of the Declaration of Helsinki.

#### Stimuli

Stimuli were normal faces or chimeric faces consisting of two half-faces belonging to different people. In order to construct our chimeric faces, we selected 64 original Western European faces (32 males and 32 females) from the Minear & Park database [51]. As stated on the web site for the database [http://agingmind.utdallas.edu/stimuli], “this [database] contains a range of face of all ages which are suitable for use as stimuli in face processing studies. Releases have been signed by the participants we photographed and the faces may be included in publications or in media events”.

**Figure 1:**
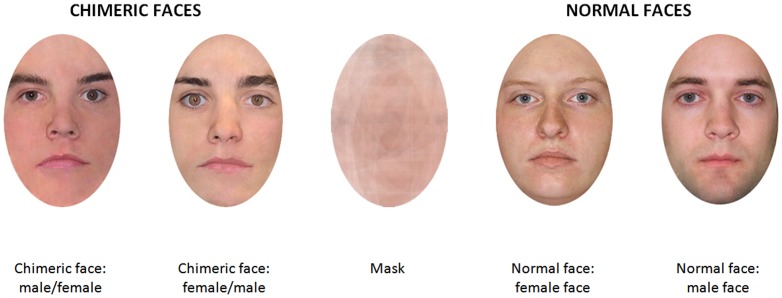
Stimuli samples and mask. Faces were selected from the Minear and Park database.

Each face was divided in two halves in a vertical plane; only one half of each face was used and associated with another half from the opposite gender. It resulted in 32 chimeric faces (see Figure 1 for examples): 16 with a female left-side face and a male right-side face, and 16 with the reverse configuration. Thirty-two normal faces were also presented: 16 males and 16 females. Finally, to avoid any effect of possible differences between the two sides of the face, we also presented the mirror image of all the 64 faces. Forty other normal faces (20 males and 20 females) were used for a pre-experiment that was designed to select the face presentation time used in the fixation condition during the experimental phase (see procedure).

All faces were in color with neutral expressions and they were cut out so that no hair or jewelry was visible. They were 8-cm-wide and 12-cm-high, and they were viewed at a distance of 57 cm, thus matching 8°*12° of visual angle. A mask with the same dimensions was made with a superposition and mix of all the faces (see Figure 1).

#### Apparatus

Chimeric and normal faces were displayed on a 19″ VGA Iiyama (HM240DT) monitor, with a spatial resolution of 600*800 pixels and a vertical refresh rate of 170 Hz. The experimental session took place in a dimly lit room. A chin and forehead rest was used in order to reduce head movements. Eye movements were recorded with an Eyelink 1000® (SR Research, Ontario, Canada) with a temporal resolution of 1000 Hz and a spatial resolution of 0.25°. Viewing was binocular but only the movements of the right eye were monitored. Each session began with a nine-point calibration over the entire screen. Before each trial, central fixation was checked and compared to the calibration. When the distance between the fixation check and the calibration was greater than 0.75°, the fixation was refused and a new calibration was initiated. When a successful calibration was detected, the trial began. Online saccade detection corresponded to the above-threshold velocity (30°/s) and acceleration (8000°/s^2^).

#### Procedure and Design

Here, we displayed normal and chimeric faces. Subjects had to perform a gender judgment task by remaining fixated on the center of the screen or by exploring the face using a unique saccade. One of our hypotheses being that executing a saccade would increase the performance compared to remaining fixated, we began the session by a pre-experiment in which we adjusted the time of face presentation in the fixation condition to avoid chance performance and ceiling effects. Thus, we used four blocks of ten trials each with only normal faces presented in top position. The experimenter gave a first presentation time (150 ms) and increased or decreased it (by variable steps depending on the initial performance) in following blocks until 80% of correct responses were obtained on two successive blocks (*M* = 203.71±17.02 ms). Generally, the presentation time reached the criteria on the second or third block and could be checked on the third or fourth block. However, if the performance was not at 80% at the 4^th^ block, the procedure was renewed. Then the experimental phase was set to begin.

During the experimental phase, four face positions could be displayed: left, right, top or bottom of the central fixation cross. Due to the shape of the face, the distance from the center of the fixation cross to the nearest edge of the face was of 1.64° for top and bottom positions, and of 3.7° for left and right positions. The distance from the center of the fixation cross to the face center was of 7.68°. Whatever the face position, participants were told to remain fixated on the cross (fixation condition) or were allowed to move their gaze, the face being off during the second saccade execution (saccade condition).

Each trial began with the fixation of a central black cross, measuring 0.5°*0.5° that was displayed for 400, 600, 800 or 1000 ms. Then, a face was presented in one of the four positions mentioned above. In the fixation condition, the mask replaced the face after the fixation time previously established in the pre-experiment. In the saccade condition, the mask was displayed when the number of allowed saccades was reached and the following saccade was detected. Participants were not aware of the restricted number of saccades (one here) as they were simply told to explore the face. In both conditions, they had to answer when the mask was displayed. Participants used a response pad with buttons marked for male and female judgment, and another button for starting the next trial. Subjects performed the task with one hand, by pressing on a button with the index finger to choose the “male” response and by pressing on the other button with the middle finger to choose the “female” response. The other hand was used to go on to the next trial. The hand response was counter-balanced across subjects.

Half of the participants were presented with the fixation block followed by the saccade block, each made of 128 trials. For the remaining participants, the order was reversed. In each block (fixation and saccade), 64 normal faces and 64 chimeric faces were presented, 16 per position –left, right, top or bottom-. Therefore, the crossing of face positions (left, right, top and bottom) and saccadic conditions (fixation or one saccade) resulted in 16 trials per condition. All faces were presented twice during the experimentation in each block. Eight lists were made including positions and faces so that every type of face (chimeric-male/female, female/male, their mirror images and normal faces) was presented at every position in each block.

#### Data Analysis

The PB was computed by subtracting the number of responses based on the right side to the number of responses based on the left side, divided by their sum. The bias varies between −1 to +1. A negative or a positive score means a left or a right bias (i.e. the participants' choices matched the gender either of the left or the right side of the face) and a zero value means that responses were equally based on the left and the right side of the face. Similarly to the PB, the GB was computed by subtracting the number of right side fixations to the number of left side fixations, divided by their sum. A negative or positive score means a left or right bias (e.g. the first saccade landed on the left or right side) and a zero value means that first fixations were equally located on the left and the right side of faces. When performing two-tailed Student T-tests for perceptual and gaze biases, we compared data to zero (i.e., absence of bias). While exploring the link between gaze and perceptual biases, we considered the gaze behavior for each participant by separating trials as a function of his/her perceptual responses, those corresponding to the left face gender and those corresponding to the right face gender.

### Results

About 10% of all trials were eliminated from further analyses for the following reasons: saccades executed in the Fixation task (6.25%), saccade with very short (<80 ms) or very long (>800 ms) latencies (2.62%), no saccade in the Saccade task (0.27%) or executed toward the wrong direction (0.82%).

#### Percentage of Correct Responses

**Figure 2:**
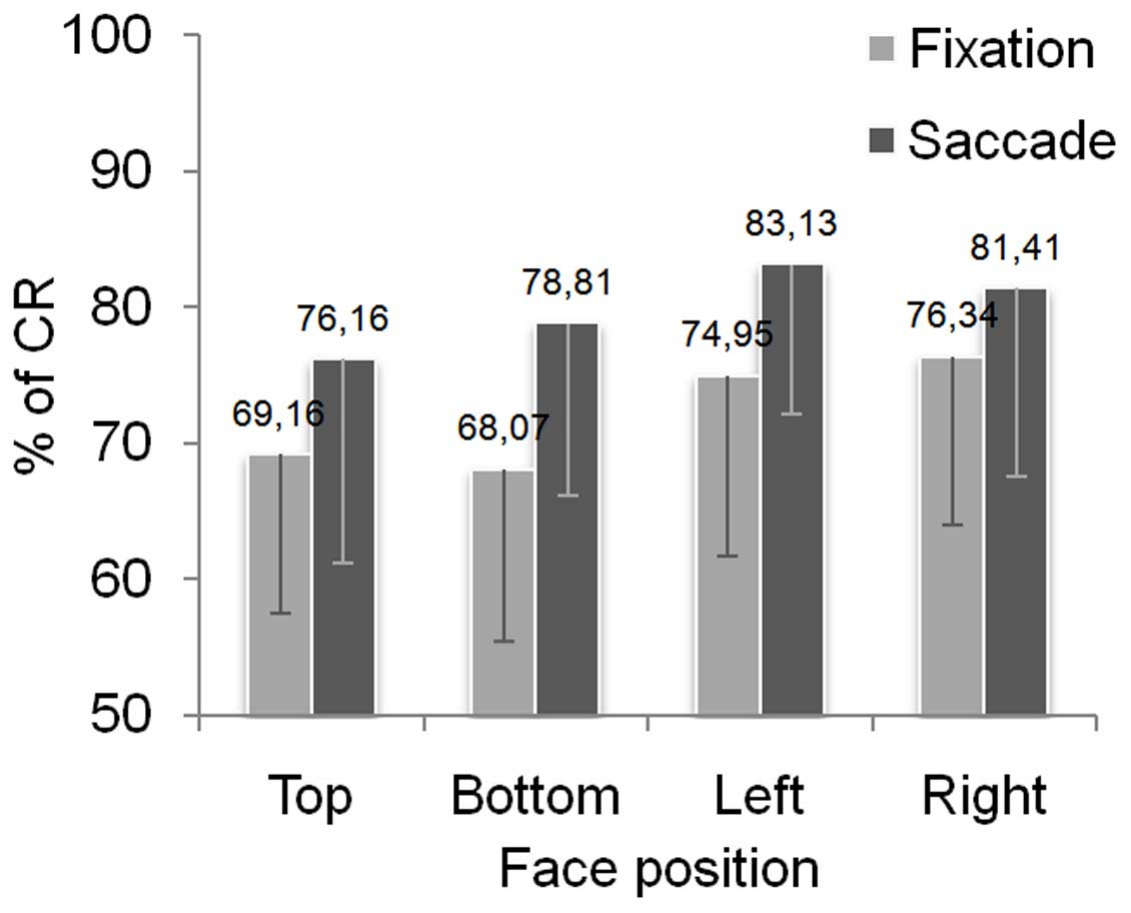
Performance in the gender judgment task for normal faces. Here are represented the % of correct responses for the fixation and saccade conditions obtained for normal faces displayed top, bottom, left or right from the central fixation cross. Error bars represent the standard deviations.

The percentage of correct responses (% of CR) was analyzed for normal faces. Overall, the % of CR was of 76% (±5.35%). As shown in Figure 2, the percentage of correct responses descriptively increases with the execution of a saccade. Moreover, the performance appears better for left and right positions compared to top and bottom positions. A first analysis was conducted to check whether our participants performed significantly above chance at discriminating gender of normal faces. We performed two-tailed Student T-Tests to compare the chance level (i.e. 50%) to the % of CR obtained in each experimental condition resulting from the crossing of face position and saccadic condition. We found that the % of CR differed significantly from chance for the four positions in the fixation condition (*t*(31) = 9.31; p<.001; *t*(31) = 8.10; p<.001; *t*(31) = 10.59; p<.001; *t*(31) = 12.03; p<.001 respectively for top, bottom, left and right position) as well as in the saccade condition (*t*(31) = 9.83; p<.001; *t*(31) = 12.82; p<.001; *t*(31) = 16.98; p<.001; *t*(31) = 12.80; p<.001 respectively for top, bottom, left and right position).

Then, an Anova was conducted with saccadic condition and face position as within-subject factors. As expected, we found a main effect of saccadic condition (*F*(1,31) = 22.93; p<.001) as the gender judgment task was best performed when one saccade was allowed compared to the Fixation condition. A main effect of face position was also observed (*F*(3,93) = 3.51; p<.05) with no interaction between the two factors (*F*<1). Planned comparisons showed that better scores were obtained for left and right positions compared to the top position (respectively *F*(1,31) = 7.30; p<.05 and *F*(1,31) = 5.07; p<.05). The comparisons with bottom position showed a difference of % of CR for right position (*F*(1,31) = 4,31; p<.05) and a marginal effects for left position (*F*(1,31) = 3.74; p = .062). Finally, no significant difference was observed on the % of CR between left and right positions (*F*<1), and top and bottom positions (*F*<1).

#### Perceptual bias (PB)

The PB provided information regarding the part of the face that was used to perform the gender judgment task; it was computed only for chimeric faces. A negative value means a left PB, when a positive value means a right PB.

**Figure 3:**
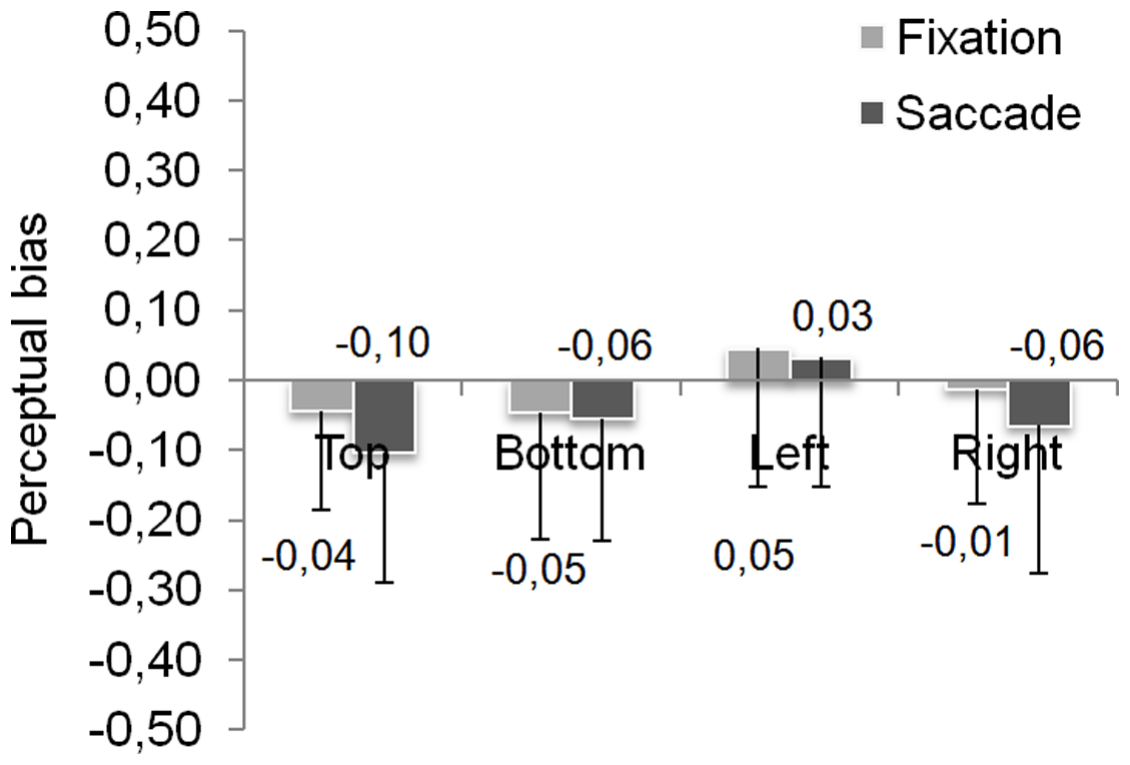
Perceptual bias values function of saccadic condition and face position. Error bars represent the standard deviations.

Figure 3 presents values of PB for each face position in fixation and saccade conditions. Overall, a slight left PB seems to emerge for fixation condition (−0.01±0.18) and then to increase for saccade condition (−0.05±0.19). Note however that the left face position seems to induce a right PB. The Anova conducted with saccadic condition and face position as within-subject factors showed a main effect of face position (*F*(3,93) = 3.61, p<.05) but no effect of saccadic condition (*F*(1,31) = 1.67, ns) and no interaction (*F*<1). Planned comparisons showed only a difference between the PB obtained in top position (−0.07±0.13) and in left position (+0.04±0.16) (*F*(1,31) = 12.81, p<.05).

We performed Student T-tests in order to compare PB value in each condition with the value of Zero (0) corresponding to an absence of bias. The PB obtained in the fixation condition did not differ from 0 (*t*(31) = −0.9; p = .35), whereas the left PB that emerged when a saccade was performed significantly differed from 0 (t(31) = −2.7; p<.05).

However, in the saccade condition, face position affected the emergence of the PB since a significant left PB was observed only for top position (*t*(31) = −4.6; p<.001). Note that the left PB differed marginally from 0 for bottom and right positions (respectively, *t*(31) = −1.8; p = .08 and *t*(31) = −1.72; p = .095). For left position, the slight right PB did not differ from 0 (t<1).

These results show that the left PB does not appear clearly in case of parafoveal presentation. Indeed, if a slight left PB seems to appear for three of our four conditions, its magnitude never differs statistically from 0. Even if the left PB seems to emerge with the initial saccade directed to the face, its magnitude differs from 0 only for top position.

**Figure 4:**
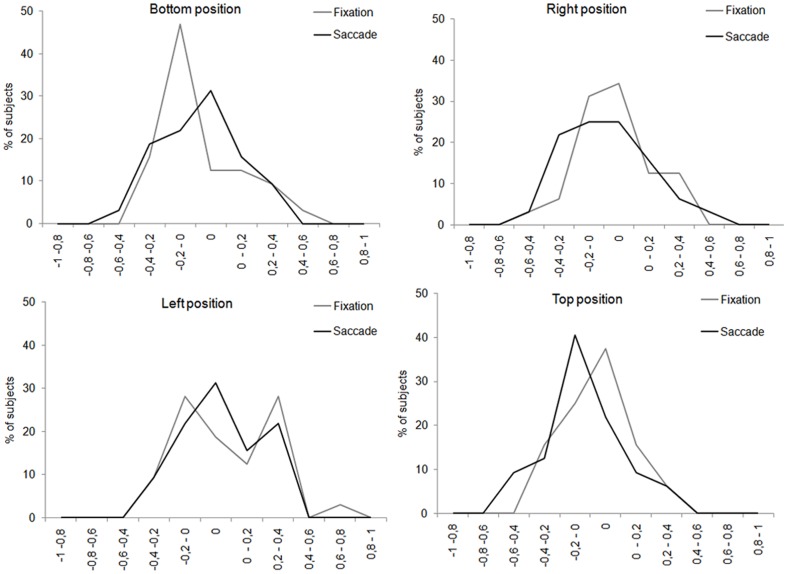
Percentage of subjects showing left right or no perceptual bias. On the abscissa, the value of the perceptual bias is plotted, with negative numbers indicating a bias towards the left side of the face and positive numbers indicating a bias towards the right side of the face. A value of 0 indicates no perceptual bias. On the ordinate, the percentage of subjects with a given value of perceptual bias is plotted.

Our results being quite different from those of previous research showing a consistent left PB for central presentation, we examined more closely the distribution of participants showing a left PB or a right PB for each position of presentation and each saccadic condition (Figure 4). We found that most subjects had a clear PB towards one or the other visual field. However, two observations must be made: first, in case of left or right PB, the magnitude of the bias is quite small, mostly under the absolute value of 0.4. Second, much to our surprise, a non-negligible proportion of participants did not show any PB, meaning that their responses to the gender task were not biased towards one or the other visual field.

#### Gaze bias (GB)

Similarly to the PB, we also examined the possibility of a GB providing information as to which part of the face was first fixated. It was computed for both types of faces, normal and chimeric as chimeric and normal faces were explored similarly (F<1). It must be noted that a negative or positive value indicates that the first saccade tends to land on the left or right side of the face.

**Figure 5:**
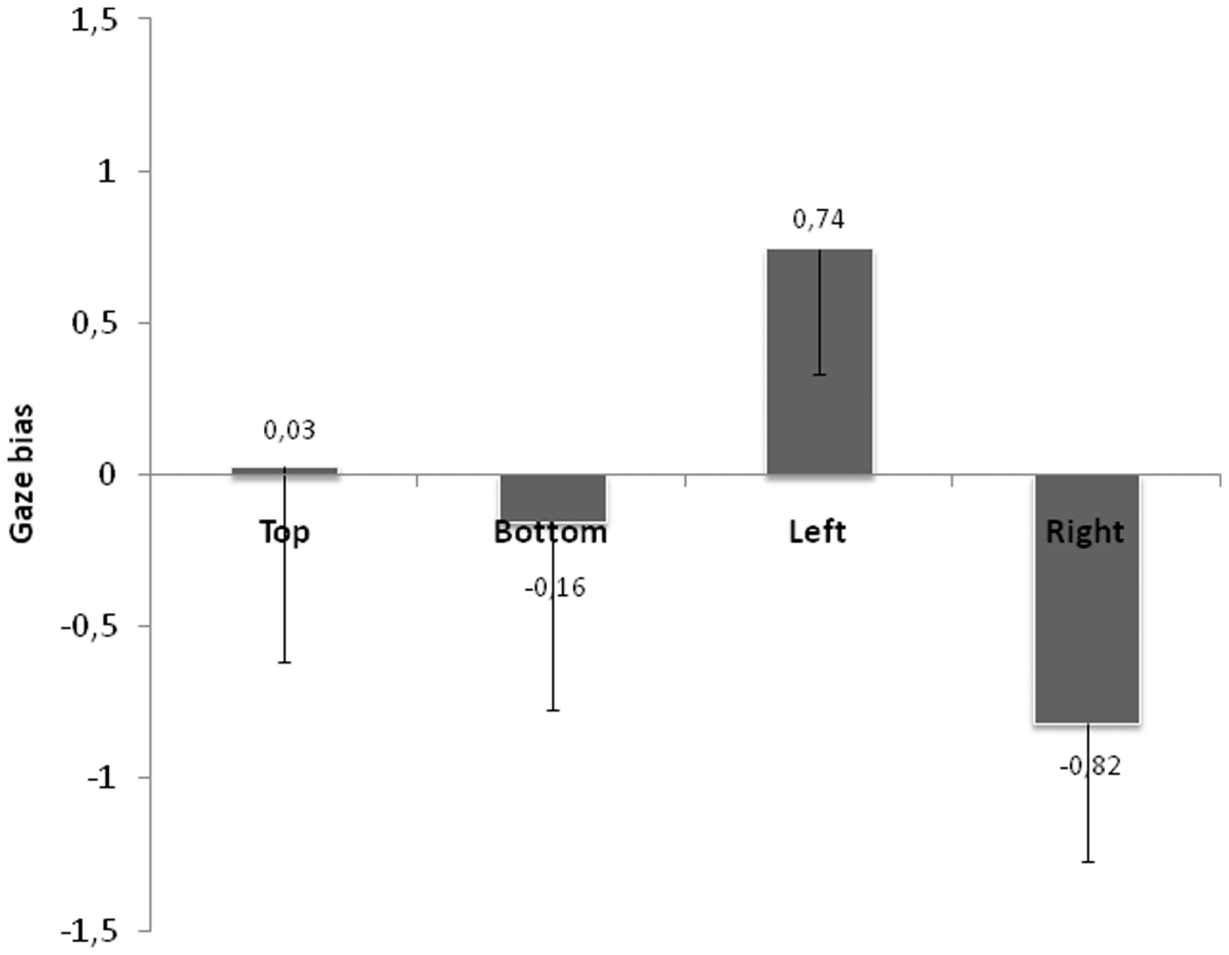
Gaze bias as a function of face position. Figure represents the gaze bias for collapsed normal and chimeric faces. Error bars represent the standard deviations.

As shown in Figure 5, the face position affects the occurrence of a GB as well as the side of the bias. Indeed, a right and left GB emerge for left and right face positions respectively. The ANOVA conducted with face position as within-subject factor showed a main effect of face position (*F*(3,93) = 64.21; p<.001). Planned comparisons showed that all comparisons were significant (all ps<.0005) except the difference between the GB obtained in the top and bottom positions (*F*(1,31) = 1.91; ns). The Student T-tests that compared the value of the GB with the value of 0 (absence of bias) showed that no clear GB appeared for top (*t*<1) and bottom (*t*(31) = −1.55; p = .13) positions. However, a GB toward the right side of the face was shown for the left position (*t*(31) = 15.90; p<.001) as well as a GB toward the left side of the face for the right position (*t*(31) = −11.51; p<.001).

**Figure 6:**
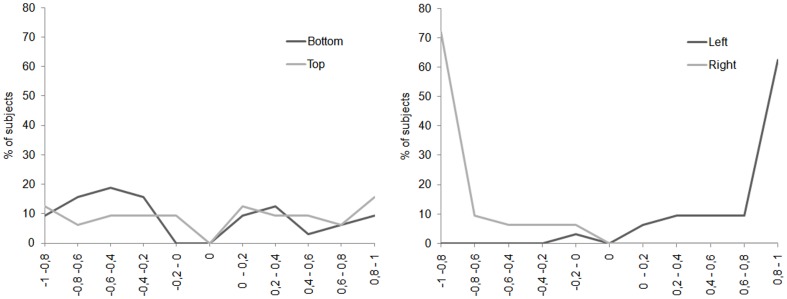
Percentage of subjects showing left right or no gaze bias. On the abscissa, the value of the gaze bias is plotted, with negative numbers indicating a bias towards the left side of the face and positive numbers indicating a bias towards the right side of the face. A value of 0 indicates no gaze bias. On the ordinate, the percentage of subjects with a given value of gaze bias is plotted.

Figure 6 presents the percentage of subjects showing a left, right or no GB. For clarity's sake, we separated the top/bottom conditions from the left/right ones. For these latter, it appeared clearly that all subjects (except one) showed a right GB for the left position and a left GB for the right position, meaning that the initial saccade always landed respectively on the right or left side of the face for the left and right position of presentation. For top and bottom positions, data were less clear since participants were distributed over all the possible range of bias, except for the value of 0.

#### Relation between gaze and perceptual biases

The above results do not establish any direct link between gaze and perceptual biases. Indeed, for left and right position of face presentation, a very clear GB appears since the saccade lands on the closest side of the face whereas no clear PB emerges. In contrast, the only condition in which a slight PB emerges, i.e. the top position in saccade condition, does not show any preference towards one or the other hemi-field for the direction of the saccade.

**Figure 7:**
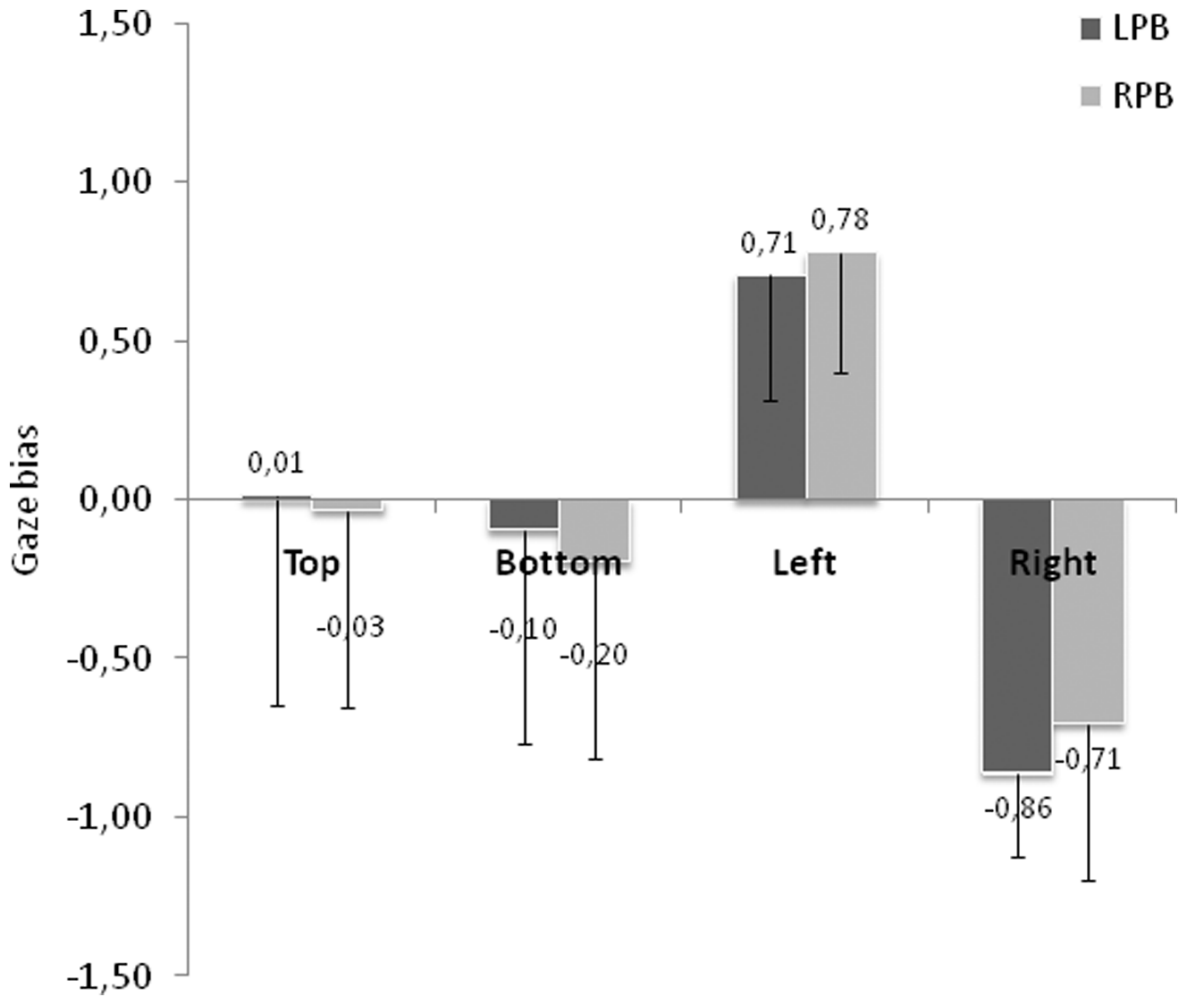
Gaze bias as a function of perceptual responses. With LPB = Left Perceptual Bias and RPB = Right Perceptual Bias. Error bars represent the standard deviations.

However, previous studies suggested that GB could vary as a function of PB [14], [42], therefore, we examined GB as a function of perceptual responses through two different analyses. In the first one, we looked at, for each face position, the correlation between the PB and the GB by taking for each participant his/her average BP and his/her average GB. None of the coefficients of correlation obtained were significant (Top position: 0.07, *t*(30) = 0.39, ns; Bottom position: 0.16, *t*(30) = 1.28, ns; Left position: 0.17, *t*(30) = 1.38, ns; Right position: 0.05, *t*(30) = 0.39, ns). This first analysis argues against a link between the PB and the GB. However, based on the Butler et al's analysis [14], we further examined a potential link between PB and GB by separating for each participant, trials as a function of the perceptual response, those corresponding to the left face gender and those corresponding to the right face gender. Then, we examined whether the gaze behavior was different. Indeed, although some participants had an average PB value close to 0, their responses to each trial were biased to the left or right hemiface. Then, the second analysis allows studying more precisely the link between PB and GB. Figure 7 clearly shows that GB does not depend on perceptual responses, as similar GB values are observed for perceptual responses biased to the left hemiface or to the right hemiface for each position. The ANOVA conducted with face position and perceptual responses as within-subject factors revealed a main effect of face position (*F*(3,93) = 56.66; p<.0005) with no effect of recorded perceptual responses (*F*<1) and no interaction (*F*(3,93) = 2.56; p<.06). The Student T-tests confirmed that the gaze pattern were similar for left perceptual responses and right perceptual responses: no GB was observed for top (*t*<1 for left perceptual responses and right perceptual responses) and bottom (left perceptual responses: *t*<1; right perceptual responses: *t*(31) = −1.8; p = .08) positions. There was a right GB for left position and a left GB for right position, regardless of the perceptual responses (for left position: left perceptual responses: *t*(31) = 10.1; p<.001; right perceptual responses: *t*(31) = 11.6; p<.001; for right position: left perceptual responses: *t*(31) = −19; p<.001; right perceptual responses: *t*(31) = −8.1; p<.001). Thus, the first saccade was not systematically oriented toward the side of the face from which subjects made their decision.

#### First landing positions and Regions of interest

**Figure 8:**
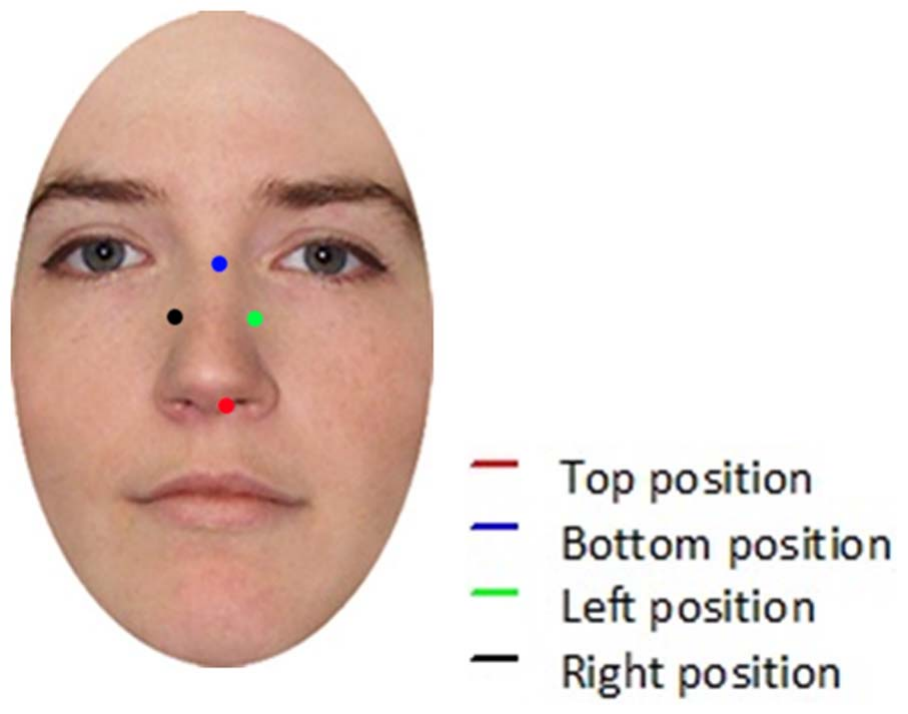
Average saccade landing positions for normal and chimeric faces collapsed. Faces were selected from the Minear and Park database.

It should be noted that the GB calculation reflects the number of times the saccade lands on the left or right hemiface without giving any information on where the saccade precisely lands on it. Figure 8 presents the average landing position of the saccade directed to faces presented left, right, top or bottom. Whatever the position of face presentation, the gaze lands around the middle of the face, very close to the vertical axis for top and bottom positions, on the right side of the face for the left position and on the left side of the face for the right position. The average X-coordinate from the midline of the stimulus was calculated for each face position in order to measure the magnitude of the eye's deviation with respect to the stimulus center. Note that a negative X-coordinate indicates a landing position left to the stimulus center whereas a positive X-coordinate indicates a landing position right to the stimulus center. An Anova was conducted with face position and type of face as within-subject factors. The main effect of face position was significant (*F*(3,93) = 96.19; p<.0005) with no effect of type of face (F<1) and no interaction (F<1). Only the difference between the landing position of the saccade for top and bottom positions failed to reach significance (respectively 0.01°±0.26° and −0.11°±0.35°, *F*(1,31) = 3.92; p<.06). All others comparisons were significant (left position: 0.91°±0.65°; right position: −0.93°±0.44°, all F(1,31)>68.93; p<.0005).

**Figure 9:**
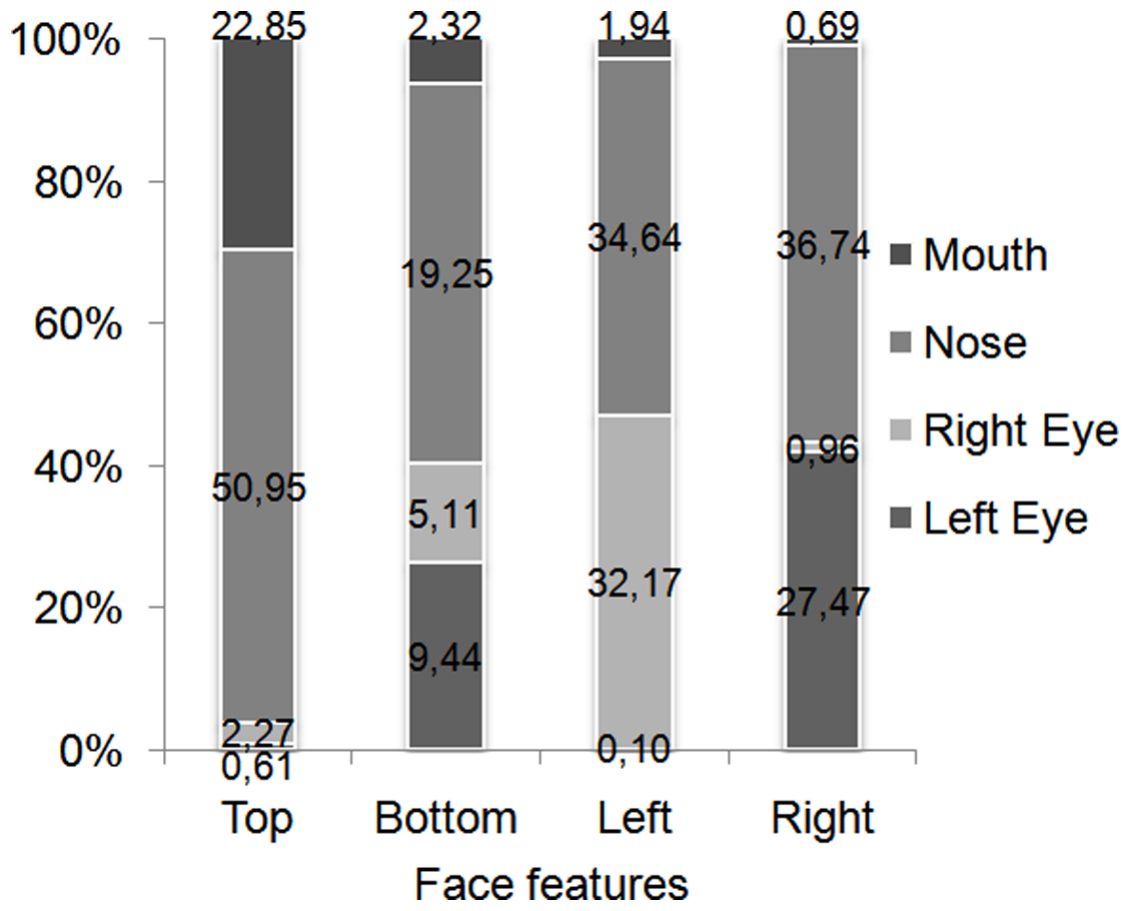
Percentage of landing position in terms of face position and regions of interest.

To further examine the landing position of the saccade, we defined six regions of interest (RoI): left and right side of the face, left eye, right eye, nose and mouth. We examined the average number of fixations on each RoI. For both normal and chimeric faces, most fixations were achieved toward the nearest side of the face when faces were displayed on the right or the left of the fixation cross. For instance, when the face appeared on the right side, most first saccades were toward the eye of the left side of the face and around the nose. When faces were displayed on the left side, fixations landed near the right eye and the nose. When faces were displayed above the fixation cross, the nose and the mouth were more frequently targeted. Finally, when a face appeared below the cross, more saccades were oriented toward the eyes and nose region (see Figure 9).

#### Saccadic latencies

The average latency of the saccade directed to bottom face position (*M* = 198.6±29 ms) was longest compared to top, left and right positions (respectively, *M* = 186.2±29 ms; *M* = 185±29 ms; *M* = 183.8±25 ms). The Anova conducted with position and type of faces as within-subject factors revealed that latencies did not vary with type of face (*F*<1), but varied with face position (*F*(3,93) = 9.89; p<.001), with no interaction between the factors ((*F*(3,93) = 2.56; p<.07). The absence of main effect of face type indicates similar processing for those two types of face stimuli which validates our chimeric stimuli.

The analysis of face position effect with planned comparisons revealed that only latency for bottom presentation differed from that of other face positions. The longest latencies for bottom presentations compared to top presentations (*F*(1,31) = 13.10; p<.001) were coherent with previous studies showing quicker latencies for saccades directed to the upper visual field than those directed to the lower visual field [52], [53]. Moreover, saccades directed to the bottom positions were also longer than saccades directed to the left (*F*(1,31) = 12.42; p<.05) and to the right positions (*F*(1,31) = 18.66; p<.001). All other differences were not significant (Fs<1).

### Discussion

In this first experiment, we examined the perceptual processing of faces and the associated gaze behavior by manipulating the initial parafoveal position and the number of saccades allowed performing a perceptual decision about gender.

For normal faces, the execution of a saccade yielded better scores to the judgment gender task compared to the fixation condition. This was consistent with previous research showing better performance in a lexical decision task in which participants had to take a decision on verbal material in fixation and saccade conditions similar to the ones used here [54]. Moreover, the gender judgment was better performed for faces that were presented either on the left or on the right compared to top and bottom positions. In the fixation condition, we expected better perceptual scores when faces were displayed on the left of the fixation cross and thus projected to- and processed by- the right hemisphere than when they were displayed on the right. Indeed, previous divided visual field studies have shown that facial recognition is better achieved in the left than in the right visual field (see [55] for a review). However, the gender judgment task differs from a recognition task as it may involve a different processing of the face which is mostly based on the analysis of distances between facial features. Indeed, several distinctive features of gender have been given such as the eye-eyebrows distance, the brow-lid distance, the nose-mouth distance as well as the chin length or the thickness of the lower lip (e.g. [56], [57]). Thus, holistic processing might not be efficient enough to perform the task and indicates that featural processing may also be required. In our experiment, one can argue that for faces presented left or right, the participants gathered the same amount of information about the distinctive facial features for gender that were mainly based on vertical distances. Indeed, the distance between the initial fixation cross and the face was similar for the two positions. However, for top and bottom presentations, the eccentricities of these different vertical distances differed and might disrupt the processing of some of these facial features (for example, in the bottom presentation, the brow-lid distance was closer from the initial fixation point than the chin length while the reverse was true for the top presentation). Consequently, the discrimination of the gender may be more difficult for top and bottom presentations than for left and right ones. Even if the execution of a saccade increased the performance, it did not eliminate the effect of the face position. This suggested that the processing of a face initially presented in the left or right visual field was subsequently facilitated during the fixation on the face, compared to top and bottom positions, and that vertical distance was pre-processed during the latency of the first saccade.

With chimeric faces, we tested whether the left perceptual and gaze biases found with central presentation of faces could be replicated with parafoveal presentation and whether they increased with the execution of a saccade toward the face. Concerning the perceptual responses, we failed to find a clear left PB for both conditions: fixation (no PB) and one saccade (left PB). Even if it appeared descriptively on three of the four conditions, its magnitude differed significantly from 0 only for the top position of presentation after the saccade landed on the face. One could argue that the small magnitude of the PB might simply reflect the fact that our participants did not process the facial information well at all. One argument against this view came from the results about perceptual performance on normal faces that was always significantly above chance. This indicates that our participants have correctly processed the facial information to discriminate gender even for parafoveal presentation. For chimeric faces, they should be also able to discriminate the gender of both hemifaces, or at least the gender of the least eccentric hemiface. Therefore, we could get an artificial perceptual bias of proximity. So, one may wonder why we did not found a larger left perceptual bias. The analysis of individual patterns gave a first explanation by showing that for bottom, top and right positions in both fixation and saccade conditions, a great number of participants had a left PB (from 41% until 62.5% as a function of the experimental condition). However, a non-negligible proportion of participants did not present any PB (from 12.5 to 37.5%), meaning that they based their responses to the gender task either on the left or right side of the face without any preference. To our knowledge, only two studies [38], [58] have reported a lack of perceptual bias for some of their participants. Finally, a smaller proportion of subjects showed a right PB (from 8% to 25%). Note that for the left position, most of the participants presented a right PB (44% and 37.5% respectively for fixation and saccade conditions). This final analysis clearly shows that the overall PB should not be interpreted without taking into account the proportion of subjects showing left, right and even no PB, as well as its magnitude. Indeed, the magnitude of the average PB clearly depends of the magnitude of the individual's PB. Some participants presenting a large perceptive bias may be enough so that the average PB becomes significant.

Our results also showed that the execution of a saccade towards the face did not increase significantly the left PB, except for the top position, and even decreased it for the bottom and the left positions. Such results did not establish any strong link between perceptual and gaze biases. This was confirmed by the analysis of the ocular behavior relative to the PB that showed no differences in the pattern of visual exploration of the face in case of perceptual responses based on the left or the right side of the face. Whatever the PB, for left and right positions, the saccade directed to the face landed on the closest side of the face relative to the initial fixation point. No clear gaze direction preference appeared for top and bottom positions, and no link with the PB was found.

This suggests that saccades are rather due to visual and oculomotor constraints, landing on the center of gravity of the faces [11] than to a direction preference linked to hemispheric asymmetry. Indeed, the analyses on the saccade landing position showed that regardless of the face position, the saccade directed toward the face landed on a position around the middle of the face, inducing a clear GB for left and right positions but not for top and bottom positions. As the average saccade landing positions differed between left and right positions compared to the top and bottom ones, we conclude that as suggested by Arizpe et al [18], the landing position was close to the center of the face but deviated toward the starting position by the combined center-of-gravity and range effects.

It may be possible that we failed to find any clear PB and even GB due to the parafoveal presentation of the faces, necessitating an initial saccade directed to the faces and/or because insufficient time to process the face in an attempt to perform the gender judgment task. Indeed, previous research has shown that the longer the face exposure duration, the more important the PB is [43]. In the second experiment, we proposed three different face positions, the two lateral from experiment 1 (left and right), and, in order to compare our results with previous studies on PB, the central position, commonly found in previous research. Participants were also given more time to explore and process the face as they were able to achieve up to three saccades before making their perceptual decision.

## Experiment 2

### Methods

#### Participants

Thirty-two young students (16 males and 16 females, *M* = 22.46±2.3 years, different from experiment 1) from Paris Descartes University, all Western Europeans and right-handed (*M* = 93.11%±5.4%), took part in this experiment. They were submitted to the same laterality and neuropsychological tests as in the first experiment and signed a consent form.

#### Procedure and Design

The materials and design were identical to those used in Experiment 1. However, here, only chimeric faces were presented. Subjects had to perform a decision gender task by remaining fixated on the center of the screen or by exploring the face (as before, participants were not aware of the restriction of the number of saccades - one, two or three here). The time of face presentation was also adjusted during a pre-experiment (*M* = 160.7±31 ms).

During the experimental phase, faces could be displayed on three positions: left, center or right of the central fixation cross. The distance from the center of the fixation cross to the nearest edge of the face was of 3.7° for left and right positions; the distance from the center of the fixation cross to the face center was of 7.68°. Whatever the face positions, participants were told to remain fixated on the cross (fixation condition) or they were told to explore the face. The face was off when the allowed number of saccades was reached and the participant initiated another saccade (saccade condition).

The fixation and saccadic blocks were made of 48 trials each: 48 trials for one, 48 trials for two and 48 trials for three saccades. Therefore, the crossing of face positions (left, central and right) and saccadic conditions (fixation, 1, 2 or 3 saccades) resulted in 16 trials per condition. Overall, participants performed 192 trials. All faces were presented three times during the experimentation. Eight lists were made including positions and faces so that every type of face (chimeric-male/female, female/male and their mirror images) was presented at every position in each block.

### Results

About 2.5% of all trials were eliminated from further analyses for the following reasons: saccades with very short (<80 ms) or very long (>800 ms) latencies (1.5%), saccades executed in the Fixation task (0.6%), no saccade in the Saccade task (0.4%), or executed toward the wrong direction (0.07%).

#### Perceptual bias (PB)

**Figure 10:**
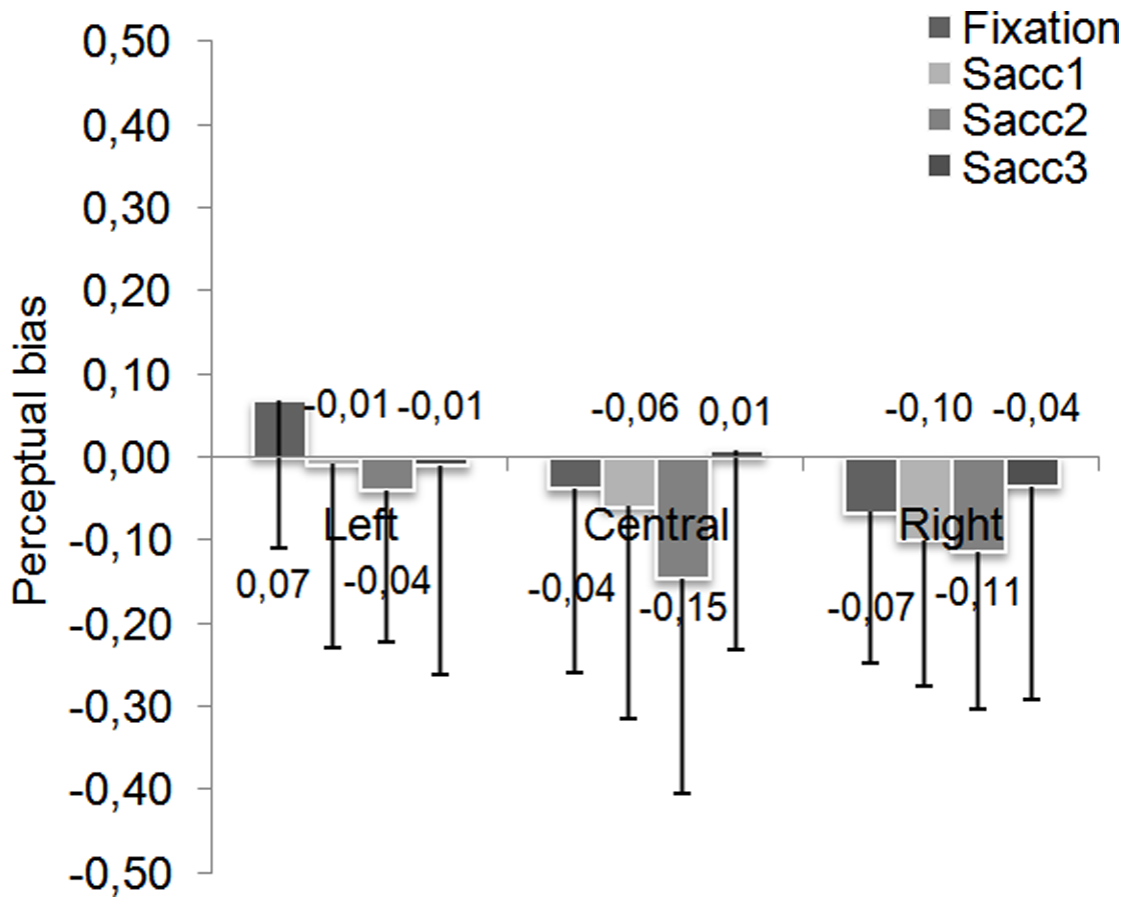
Perceptual bias values function of saccadic condition and face position. Error bars represent the standard deviations.

Figure 10 shows the PB as a function of saccadic condition and face position. Descriptively, a left PB appears for the central and right positions that increases from fixation to two-saccade conditions and disappears when three saccades are performed in the face. Note that for the left position, the perceptual responses, biased to the right side of the face in the fixation condition, appears to be biased to the left side of the face when the face can be sampled. The Anova conducted with saccadic condition and face position as within-subject factors revealed a main effect of saccade condition (*F*(3,93) = 3.62; p<.05) as well as an effect of face position (*F*(2,62) = 4.99; p<.05) with no interaction (*F*<1). Planned comparisons showed significant differences between fixation and two-saccade conditions (*F*(1,31) = 6.60, p<.05), between two-saccade and three-saccade conditions (*F*(1,31) = 11.69, p<.01), as well as significant differences between left and central positions (*F*(1,31) = 7.77, p<.01) and left and right positions (*F*(1,31) = 7.82, p<.01).

Again, Student T-tests were performed to compare the PB values to 0 (i.e., absence of bias). As in the first experiment, the PB obtained in the fixation condition did not differ from 0 (*t*<1) whereas a left PB emerged for the one-saccade condition (*t*(31) = −2.47, p<.05). Moreover, this left PB was maintained in the two-saccade condition (*t*(31) = −4.31, p<.001) but disappeared in the three-saccade condition (*t*<1). Moreover, the face position seemed to influence the PB again. Indeed, when the face was displayed on the left side of the fixation cross, a right PB was observed (*t*(31) = 2.15; p<.05) in the fixation condition whereas no PB was observed for all saccade conditions (one-saccade: *t*<1; two-saccade: *t*(31) = −1.25; p = .22 and three-saccade: *t*<1). For central position, a left PB emerged only when two saccades were performed (*t*(31) = −3.2; p<.05) but disappeared when a third saccade was allowed (*t*<1). Finally, when the face was displayed on the right, a left PB was recorded for fixation (*t*(31) = −2.09; p<.05), one-saccade (*t*(31) = −3.2; p<.05) and two-saccade conditions (*t*(31) = −3.41; p<.05). After a third saccade, no PB was noticed (*t*<1) for any of the three positions.

**Figure 11:**
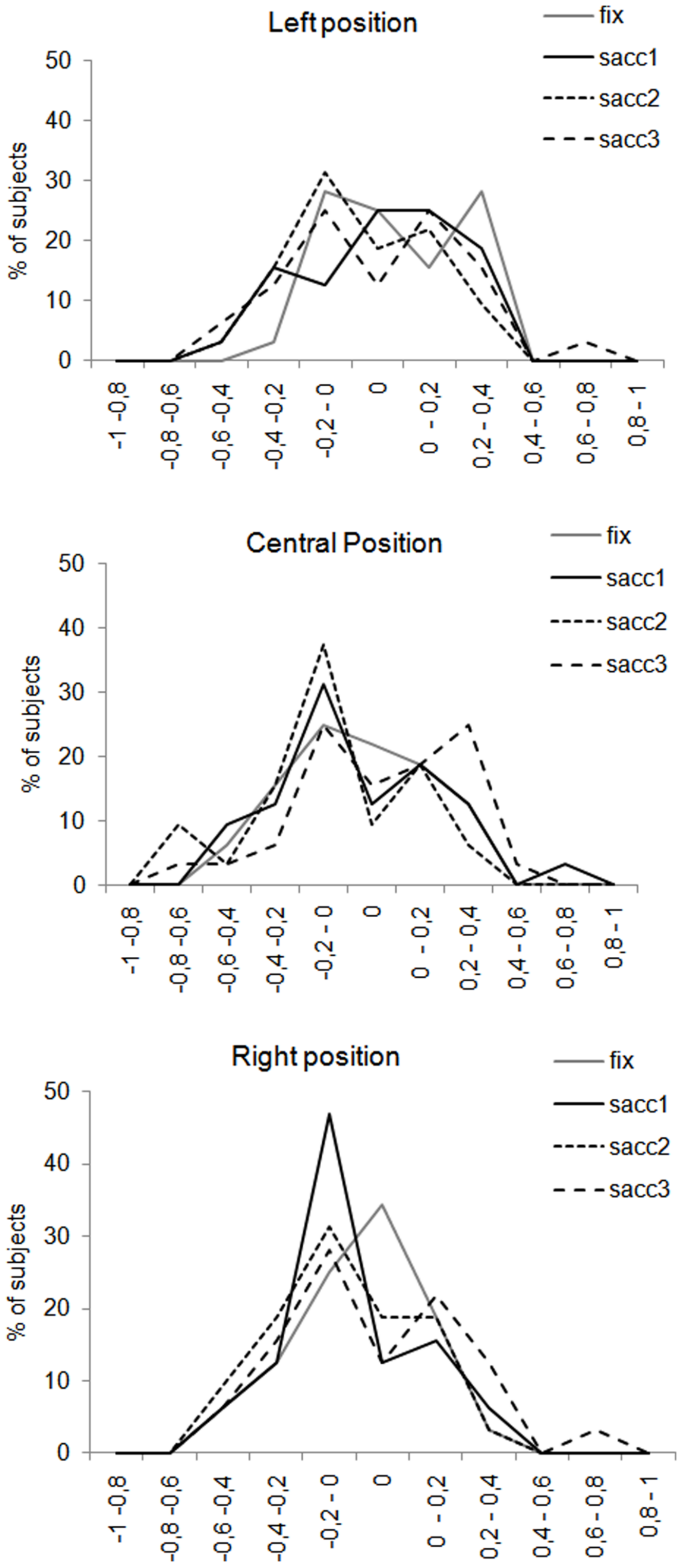
Percentage of subjects showing left right or no perceptual bias. On the abscissa, the value of the perceptual bias is plotted, with negative numbers indicating a bias towards the left side of the face and positive numbers indicating a bias towards the right side of the face. A value of 0 indicates no perceptual bias. On the ordinate, the percentage of subjects with a given value of perceptual bias is plotted.

As expected, this former analysis of the overall PB show that the left PB increased when the participants were able to explore the face. Although the left PB gradually increased from the fixation condition until the two-saccade condition, however, it disappeared in the three-saccade condition. A closer examination taking into account the position of the face showed that the left PB differed significantly from 0 only in the two-saccade condition for the central position and up to the two-saccade condition for the right position. As in the first experiment, we examined the distribution of participants showing a left/right or no PB (Figure 11). For central and right positions, most of the participants presented a left PB in the vast majority of the saccade conditions whereas for the left position, the participants were more distributed around all the range of PB. Again, as in the first experiment, a non-negligible proportion of participants did not show any preference for one or the other hemi-field to make their perceptual decision.

#### Gaze bias (GB)

**Figure 12:**
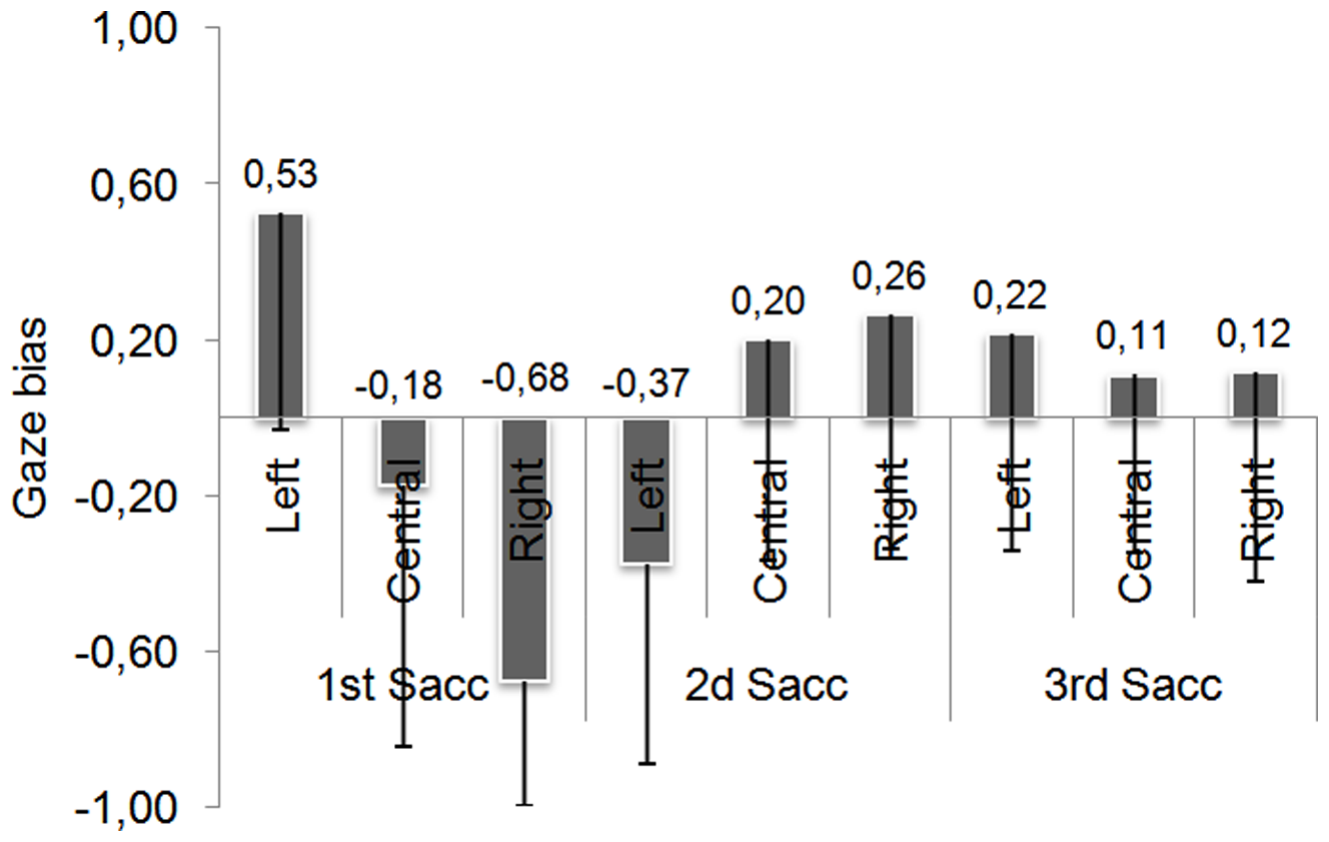
Gaze bias as a function of face position and saccadic rank. Error bars represent the standard deviations.

Figure 12 presents the GB obtained on the first, second and third saccade for each face position. The GB seems to be affected both by the face position and the rank of the saccade. The Anova conducted with face position and saccade rank as within-subject factors confirmed the main effects of face position (*F*(2,62) = 11.68; p<.001), of saccade rank (*F*(2,62) = 8.63; p<.001) as well as the interaction between the two factors (*F*(4,124) = 27.33; p<.001). The interaction was explained by the absence of face position effect for the third saccade (F<1), all others comparisons being significant (ps<.01).

The Student T-tests indicated that when the face was displayed on the left, the first saccade landed on the right half-face (the closest half-face to the fixation cross) (*t*(31) = 5.31; p<.001), the second saccade was oriented toward the left half-face (*t*(31) = −4.10; p<.001) (the farthest half-face) and the third saccade returned to the right half-face (*t*(31) = 2.18; p<.05).

When the face was displayed centrally, the gaze remained around the center of the face for the first and the third saccades (1^st^Sacc: *t*(31) = −1.48; p = .15; 3^rd^Sacc: *t*(31) = 1.35; p = .18). The second saccade had a tendency to land on the right side of the face (*t*(31) = 1.99; p = .055).

Finally, when the face was displayed on the right, the saccade landed on the left side of the face (*t*(31) = −11.83; p<.001), and moved toward the right side of the face during the second saccade (*t*(31) = 2.48; p<.05). On the third saccade, the fixation was performed around the center of the face (*t*(31) = 1.23; p = .22).

**Figure 13:**
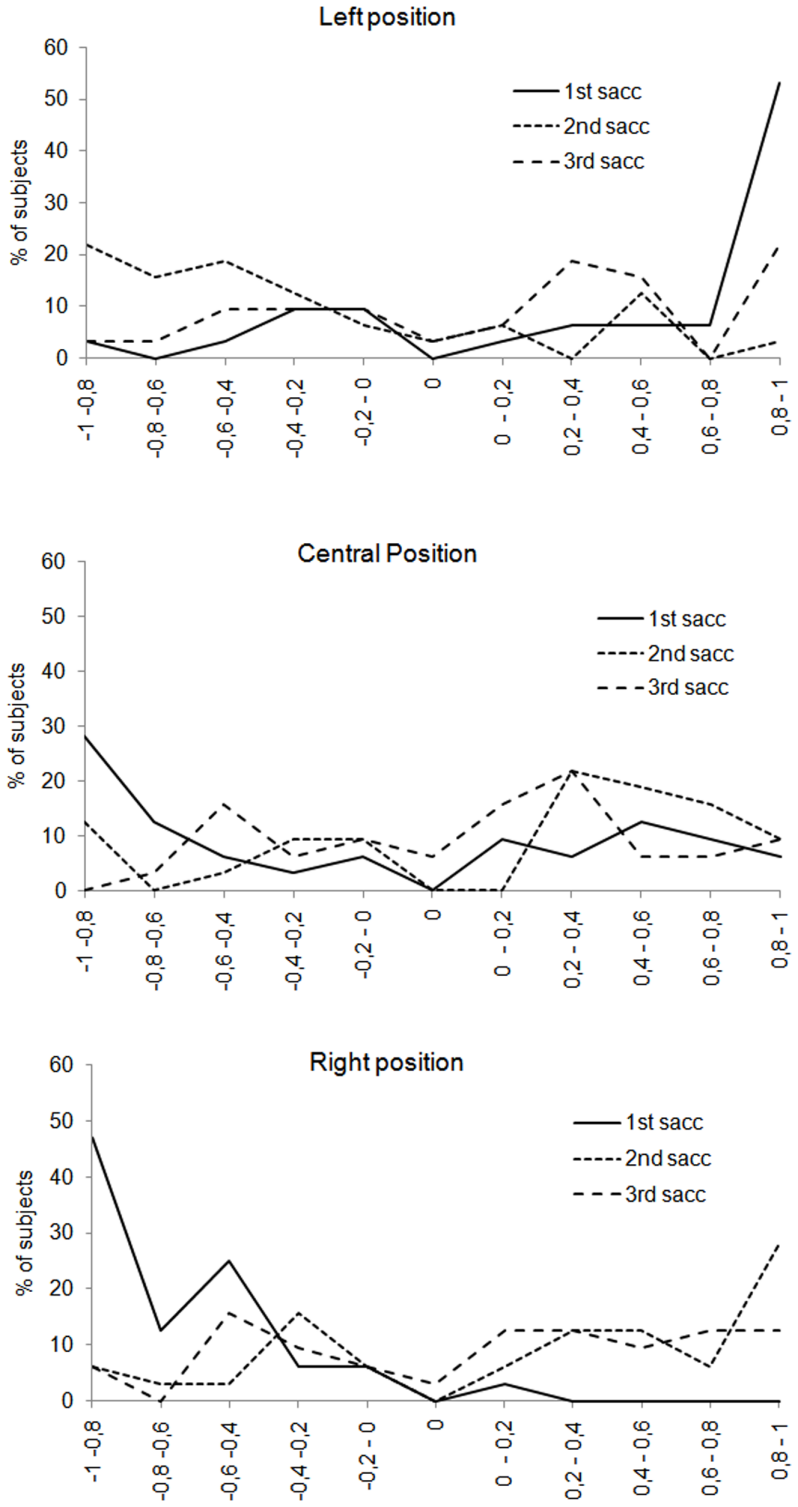
Percentage of subjects showing left right or no gaze bias. On the abscissa, the value of the gaze bias is plotted, with negative numbers indicating a bias towards the left side of the face and positive numbers indicating a bias towards the right side of the face. A value of 0 indicates no gaze bias. On the ordinate, the percentage of subjects with a given value of gaze bias is plotted.

Figure 13 presents the percentage of subjects showing a left, right or no GB. As in the first experiment, most subjects showed a right GB on the initial saccade for the left position (75% of participants) and left GB for the right position (97% of participants). For the second and the third saccades, the participants were more distributed, even though most of the participants performed a second saccade on the other side of the face. For the central position, although the average GB did not differ significantly from 0, around 56% of the participants looked initially at the left side of the face and then at the other side of the face.

#### Relation between gaze and perceptual biases

**Figure 14:**
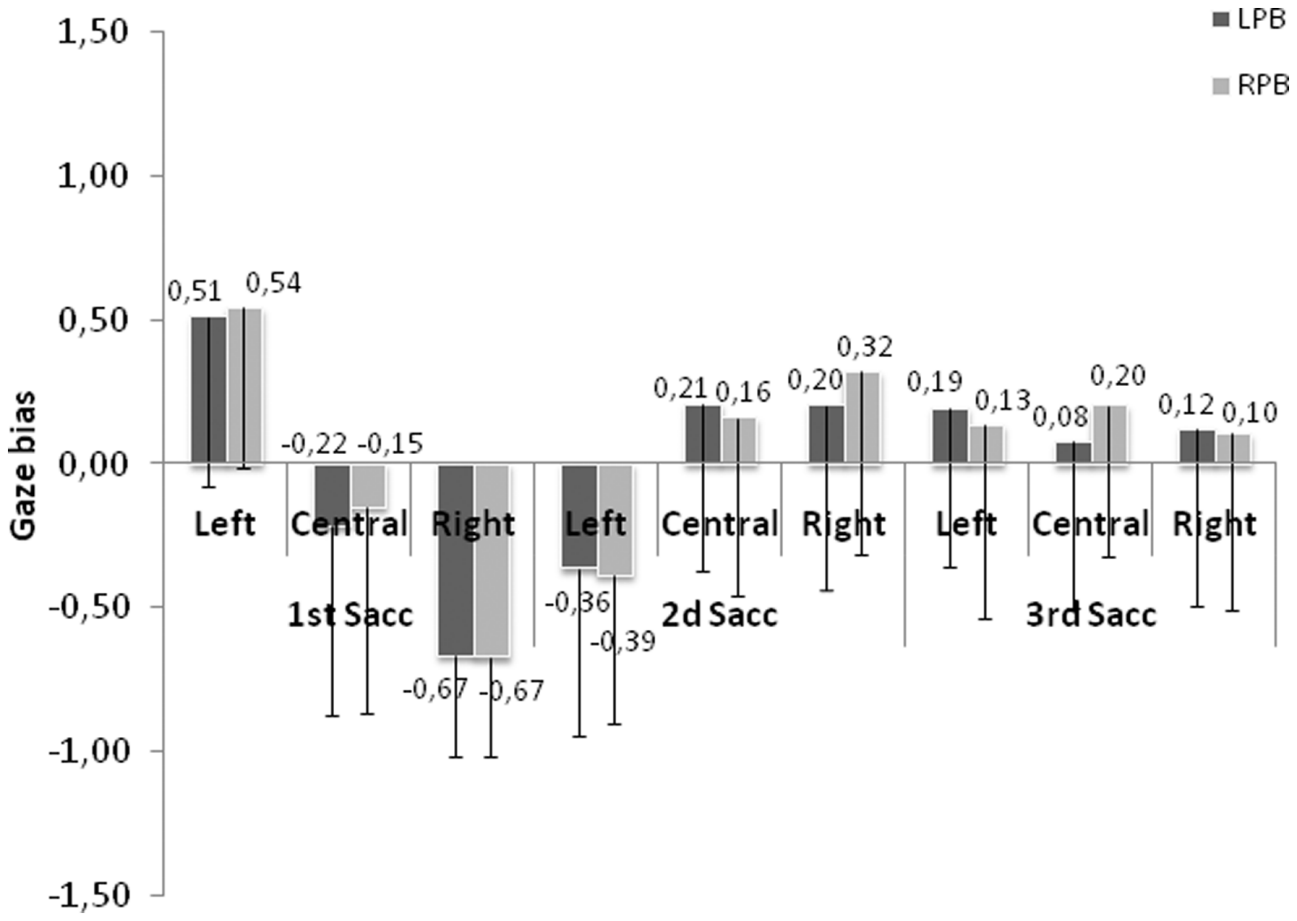
Gaze bias as a function of perceptual responses. With LPB = Left Perceptual Bias and RPB = Right Perceptual Bias. Error bars represent the standard deviations.

Similar to the analyses conducted in the first experiment, we first looked at the correlation between the PB and the GB for each face position and each saccade rank. Again, we failed to find significant correlations (coefficients of correlation between −0.12 and 0.19) except for the third saccade directed within the face initially presented to the left (coefficient of correlation of 0.49, *t*(30) = 3.13; p<.005). In the second analysis, we looked at the gaze behavior by separating for each participant the perceptual responses biased to the left or to the right. As shown in Figure 14, gaze patterns are similar for each face position and saccadic rank regardless perceptual response. The Anova conducted with face position, saccadic rank and perceptual response as within-subject factors confirmed that the GB did not depend on perceptual response (*F*<1). The main effects of face position and saccadic rank were significant (respectively *F*(2,62) = 9.94; p<.0005 and *F*(2,62) = 8.25; p<.0005) as well as the interaction between these two factors (*F*(4,124) = 26.60; p<.0001). No other interactions were significant. As for the GB analysis, the interaction was explained by the absence of face position effect for the third saccade (F<1), all other comparisons being significant (all ps<.01).

The Student T-tests revealed that for central position, no clear GB occurred on the central position for first and second saccade (respectively 1^st^ saccade: Left PB (*t*(31) = −1.87; p<.08; Right PB (*t*(31) = −1.2, ns; 2nd saccade: left PB (*t*(31) = 2.03; p<.06; right PB (*t*(31) = 1.46; ns), whereas the third saccade elicits a right GB for right perceptual responses only (*t*(31) = 2.75; p<.05) but no GB when the left side of the face was used to make the gender judgment (*t*<1). For left position, a right GB appears on the first saccade (Left PB (*t*(31) = 4.92; p<.0005; Right PB (*t*(31) = 5.52, p<.0005) followed by a left GB on the second saccade (2^nd^ saccade left PB (*t*(31) = −3.51; p<.001; right PB (*t*(31) = −4.27, p<.0005). The third saccade also had a tendency to land on the right side of the face (Left PB (*t*(31) = −1.99 p<.054; Right PB (*t*(31) = 1.12, ns). Finally for right position, the first saccade presented a left GB (Left PB (*t*(31) = −10.07; p<.0005; Right PB (*t*(31) = −11, p<.0005) whereas the second saccade landed on the right side of the face (Left PB (*t*(31) = −1.81; p<.08; Right PB (*t*(31) = −2.83, p<.008). No significant bias occurred on the 3^rd^ saccade (Left PB (*t*(31) = 1.11; ns; Right PB *t*<1).

#### Landing position and Regions of interest

**Figure 15:**
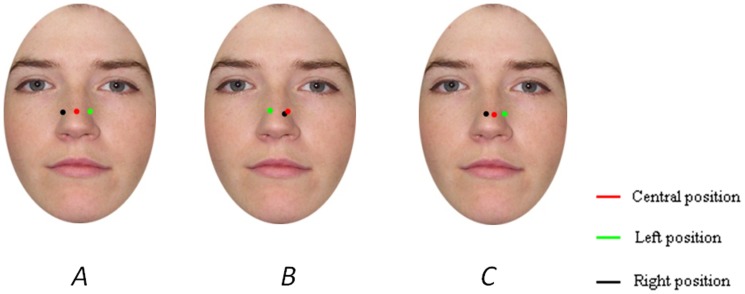
Average saccade landing positions. Data collapsed for (A) first saccade, (B) second saccade and (C) third saccade. Faces were selected from the Minear and Park database.

Figure 15 presents the average landing position of the first, second and third saccade directed to faces presented left, right, or center. When faces are displayed centrally, the average landing position is around the middle of the nose for the three saccades. For left and right positions, the first saccade lands in the closest half-face, the second in the further half-face and finally the third around the center of the face. As in the first experiment, the average X-coordinate from the midline of the stimulus was computed for each face position and each saccade rank in order to measure the magnitude of the eye's deviation with respect to the stimulus center. An Anova was conducted with face position and saccade rank as within-subject factors. The effect of face position was significant (*F*(2,62) = 11.26; p<.0005), as well as the effect of saccade rank (*F*(2,62) = 4.42; p<.05), with an interaction between the two factors (*F*(4,124) = 13.36; p<.0005). The interaction was mainly due to the fact that there was no effect of saccade rank for the central position (*F*(2,62) = 1.97; ns) but an effect for the right and left position (*F*(2,62) = 13.70; p<.0005 and *F*(2,62) = 17.83; p<.0005 respectively). More importantly for our purpose, the effect of the face position was significant for each of the three saccade ranks (*F*(2,62) = 40.49; p<.0005; *F*(2,62) = 4.58; p<.05 and *F*(2,62) = 6.36; p<.005 for the first, second and third saccade respectively). Specific comparisons showed that only the differences between landing positions of the second and third saccades for central and right presentations were not significant (respectively F<1 and *F*(1,31) = 1.77; ns). All other comparisons were significant (All F(1,31)>4.88; p<.04).

**Figure 16:**
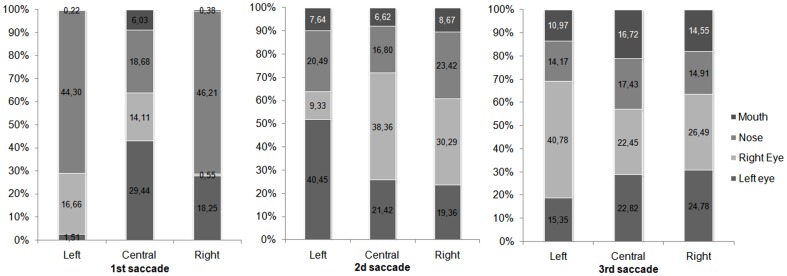
Percentage of landing positions as a function of face positions and regions of interest.

The same regions of interest (RoI) as in experiment 1 were defined here: left and right side of the face, left eye, right eye, nose and mouth (Figure 16).

For left position, the first saccade lands in the nose and right eye region. The second on the left eye and nose region and the third mainly on the right eye, but also on the nose the right eye and the mouth region. For central position, the first fixation is mainly on the left eye, or the nose, and then the second is on the right eye and similarly divided among the nose and the left eye region. Finally, the third saccade is equally divided between the right and the left eye, and then between the nose and the mouth region. For the right position, the first saccade lands in the nose and the left eye region, the second mainly in the right eye and then similarly on the nose and the left eye region. The last saccade is divided between the left and the right eye, and then equally divided among the mouth and the nose region.

#### Saccadic latencies

Latencies depend on the rank in the saccadic sequence as well as on the face position. The Anova conducted with face position and saccadic rank as within-subject factors revealed main effects of saccadic rank (*F*(2,62) = 16.31; p<.001), of face position (*F*(2,62) = 47.84; p<.001), as well as an interaction (*F*(4,124) = 10.44; p<.001).Even though the pattern of latency was similar among saccadic rank, with longest latencies for central position compared to lateral positions, the greatest difference was observed for the first saccade (central: m = 325±100 ms *vs.* lateral: m = 194±34.7 ms; *F*(1,31) = 63.95; p<.001). This difference was also observed for latencies of the second (central: m = 298±115 ms *vs.* lateral: m = 241±86 ms; *F*(1,31) = 17.17; p<.001) and third saccade (central: m = 344±107 ms *vs.* lateral: m = 309±98 ms; *F*(1,31) = 6.18; p<.05).

### Discussion

In this experiment, we compared the perceptual and gaze biases when faces were presented centrally or parafoveally in the left or the right visual field, and examined their possible evolution with the number of saccades allowed to explore the stimuli. As in the first experiment, we found that the left PB was enhanced with saccades, at least in conditions where one or two saccades were executed. Indeed, no PB appeared in the three-saccade conditions. However, as in the first experiment, the initial position of the face seemed to be a critical factor for the emergence of a left PB as it was found only for central and right positions. The analysis of individual patterns replicated on a new group of participants the fact that individual biases were distributed from left to right bias with an important proportion of participants showing no PB. Concerning the GB, we replicated the initial “proximity” GB found in the first experiment for the left and right positions as the saccade directed to the face landed on the closest side of the face relative to the central initial fixation point. For the central position, a left GB emerged descriptively but failed to reach significance. Whatever the initial position for presentation, the second saccade consistently landed on the other side of the face compared to the first saccade, whereas the third saccade had a tendency to land on the right side of the face, near its center. More importantly, as in the first experiment, the same ocular exploration of faces was found irrespective of the perceptual responses of the participants. One could argue that the GB did not differ as a function of PB because most fixations were still close to the midline of the face. However, as in the first experiment, we found that the average landing positions of the first, second and third saccades in left, right and central faces clearly differed from each other. Moreover, the fixated regions of interest differed, indicating that eye movements were not always artificially directed around the nose by the temporal constraints of our paradigm.

## General Discussion

The two experiments presented here were conducted to further examine independently the left perceptual and gaze biases commonly reported in the literature, as well as the possible relationship between these two biases. Particularly, we were interested in examining the impact of face position as well as in the influence of the number of saccades allowed to perform the gender judgment task. Across the two experiments, faces would be initially presented on a central or a parafoveal position (left, top, right and bottom of the initial fixation point) and participants had to make a gender decision on chimeric faces after exploring the face with one, two or three saccades. Our results show that the PB depends on the initial position of faces and the time allowed exploring the face whereas the GB seems mainly guided by the oculomotor and visual constraints linked to the stimuli exploration (i.e. combined center-of-gravity and range effects). They also clearly demonstrate the absence of any relationship between the perceptual and the gaze bias at a global and individual level. Finally, they pointed to the need of taking into account biases at an individual level so as to better understand the perceptual and motor processes that are involved in face processing.

Using central presentation, previous studies have demonstrated a left PB, meaning that observers generally select the left side of the face when they have to make a decision based on age, gender or emotion shown on a face (e.g. [29], [31], [33]). This left PB has been linked to the dominance of the RH for face processing, leading to a better processing of the side presented in the left visual field of the observers. However, the magnitude of the left PB depended on the task as around 55% to 78% of perceptual responses were based on the left side of chimeric faces whether the task requires a judgment of expression, gender or age [29]. It also depends on face exposure duration, a left PB being present in a gender judgment task for a presentation of only 100 ms, and then gradually increasing with the face exposure duration (until 2 seconds for Butler et al. [43]). However, to our knowledge, no studies have examined whether a left PB occurred for initial parafoveal presentation of faces. Arizpe et al. [18] suggested a highlight of the experimental bias by the commonly used center start position. Such position induces qualitatively different information processing as well as a different pattern of fixations throughout the trial different from an initial fixation point outside the face. To avoid such initial fixation bias, Petersons & Eckstein ([45], [46]) used peripheral starting locations to examine the first saccade landing position on a centrally presented face but did not make subsequent analyses on the starting position effect. Here, we have presented the face in different positions from the initial fixation point -left, right, top and bottom. The top and bottom positions always induced a presentation of the left and right sides of the faces respectively in the left and right visual fields of the participants; this was not the case for the left and right positions in which the whole chimeric face was either in the left or the right visual field. Our results show that for parafoveal vision (i.e., when the gaze remains central on the screen), a left PB descriptively emerges for top, bottom and right positions (only statistically significant in the right position of Experiment 2) whereas a right PB emerges for the left position (only statistically different from 0 in Experiment 2). Although the left PB for top and bottom positions is consistent with an explanation in terms of hemispheric asymmetry, the PB obtained for lateral positions is more consistent with what may be called a “proximity perceptual bias”, meaning that the decision about the gender of the chimeric faces was made from the closest side of the face. For the central position, the left PB that emerges descriptively was not statistically different from 0 in the fixation condition. As mentioned in the discussion of the first experiment, the small magnitude of the PB might reflect the fact that in a certain proportion of trials, participants had difficulties to determine the gender of the stimulus. However, the analyses of perceptual performance on normal faces done in the first experiment showed that the percentage of correct responses on the gender judgment task was always significantly above chance for normal faces indicating that participants were able to correctly process the facial information to discriminate gender even for parafoveal presentations. Therefore, we assume that they are also able to discriminate the gender of hemifaces of chimeric faces, or at least the gender of the least eccentric hemiface, as suggested by the “proximity perceptual bias”. Following Butler et al. [43], one may argue that faces were being presented too briefly to allow the emergence of the left PB even though our mean exposure durations were greater than the 100 milliseconds used in their experiment (mean times of 200 ms and 160 ms for first and second experiment). However, in conditions in which participants explored the faces with up to three saccades, we found that except for the left position, the exploration of faces with one or two saccades globally increased the left PB, even though the significance was not reached in all conditions. Then, the PB disappeared with the third saccade. Such results show that the left PB may depend on the face exposure duration or by the possibility to explore visually the face. In the daily life, both factors were confounded and our experiments were not designed to disentangle them.

However, we wonder why we failed to find a clear left PB in the commonly used central position in particular for the fixation and one-saccade. Indeed, in most of studies, the left PB was found [29], [41], even for short duration of presentation [43]. The examination of individual profiles revealed that although most participants presented the expected left PB, some of them exhibited a right PB. This was consistent with previous studies that also pointed out a right PB in some subjects. For instance, Butler & Harvey [42] reported that 23% of their subjects exhibited a right PB, Yovel et al. [38] a proportion of 35%. The most surprising finding in our experiment was that the non-negligible proportion of participants that did not present any preference for one or the other side of the face. To our knowledge, only two previous studies indicated the presence of some participants without PB [38], [58]. Several hypotheses may account for our result. Saether and collaborators [19] suggested that maybe in a gender judgment task, the right side of the face was “as equally important as the left side of the face” or more probably that “individuals largely differ in their perceptual biases”. Our results confirm the latter statement. Additional arguments came from the fMRI study of Yovel et al. [38] establishing a correlation between the behavioral PB and the asymmetrical activation of the Fusiform Gyrus. So, even though we were not able to test such hypothesis, it is likely that PB profile for each observer was linked to the profile of activation of areas related to face processing. Note however that this cannot be the only explanation as to why we failed to find a consistent intra-individual PB, meaning that for each of our participants, the PB was clearly dependent on the face position: the PB appeared to be a combination of stimuli-driven and internal characteristics.

Independently from studies looking at PB, a left GB has been also reported, meaning that during face exploration, the gaze was directed preferentially to the left side of the face [16], [20], [22]–[24]. Such left GB was reported on the initial saccade directed to or within the face, as well as on the total proportion of fixation and total inspection time. As for the left PB, it has been linked to the right hemisphere dominance for face processing. The RH receiving inputs from the left visual field, it has been suggested that the gaze was directed to the more “salient” side of the face [14], [30]. However, as noticed by some authors, in terms of RH advantage hypothesis, it would be more advantageous for the system to direct the initial saccade to the right side of the face in order to maintain the left side of the face in the left visual field (e.g. [44]). The question remains whether the face exploration is an expression of hemispheric specialization or an expression of perceptual experience and oculomotor constraints linked to face processing. Indeed, Bindemann et al. [11] have demonstrated that the initial saccade directed to a face is driven by general stimulus properties, landing on the center of gravity of the face whereas subsequent eye movements are directed to interesting specific facial features such as the eyes or the nose. In a similar way, Saether and collaborators [19] have shown that during a gender judgment task, the gaze is directed within an “infraorbital region” of the face, irrespective of the face's angle of view. They proposed a gaze strategy in which a central “anchor point” was selected, biased towards an intermediate position between the eye and the nose in front, and gradually more between the eye, nose and cheek as the head turns. The authors proposed that such a positioning of gaze might be optimal for the perceptual task involved here. For frontal views, this is coherent with the results of Hsiao & Liu [44] showing that the optimal viewing position for face recognition is slightly left of its center, between the nose and the cheekbone of the face. However, their experimental procedure using an imposed initial fixation on the face differed from the natural gaze exploration of face. By using two conditions, one involving free eye movements during a face-identification task and another restricting the gaze to specific locations on face, Peterson and Eckstein ([45], [46]) confirmed that observers moved their eyes to a location just below the face's eyes that maximized perceptual performance during face processing. The results on the GB in our experiments are in favor of a visual exploration linked to stimuli properties more than hemispheric specialization. Indeed, we replicate an initial landing position near the face center similar to previous studies [11], [19], [21] which clearly depends on the initial position of presentation. Whatever the face position, the first saccade was oriented toward the closest half-face, (i.e. the right half-face for the left position, the left half-face for the right position, the lower half-face for the top position and the upper half-face for the bottom position). This “proximity” bias for parafoveal positions leads to initial fixations around the face center for each of the four possible face positions. So, the consistent right GB for the left position and the left GB for the right position are only due to the fact that the initial saccade lands on the closest side of the face in eccentricity. Such results are consistent with Armann & Bülthoff [13] that have recorded eye movements in gender and identity comparison tasks of faces presented simultaneously. They found that observers compared predominantly the inner halves of the face stimuli, a result which is inconsistent with the left GB generally reported for presentation of a single face. Here, we show the same effect with presentation of a single parafoveal face, suggesting that the effect is not task-dependent but linked to the aim of the center of gravity of faces combined with the tendency for saccades to undershoot near targets [18]. For top and bottom positions, the initial landing position was so close to the boundary between left and right side of the face that no clear left GB appears on the first saccade. For central position, a descriptive left GB emerges but fails to reach significance. Moreover, as for the PB, the analysis of individual patterns clearly shows that the participants were distributed over the entire range of possible GB.

A second argument in favor of visual exploration linked to perceptual and oculomotor constraints is that, as suggested by Arizpe et al. [18], the initial starting position influences subsequent fixations patterns. Indeed, the second saccade landing position depends on the initial saccade position. The second saccade reaches a position on the other side of the face compared to the first one. Note that the third saccade is likely to land on the right side of the face but the magnitude of the bias is smaller than for first and second saccades. So, the analysis of the visual exploration of faces in the gender judgment task suggest that observers move their eyes from one side to the other with saccades landing near the face center, including fixations on nose, eyes and mouth. Again, this was coherent with previous studies [11], [19], [21]. Please note however that our gender judgment task may have contributed to limit the left GB commonly found with central presentation as participants had to manage with conflicting gender cues provided by chimeric faces. Nevertheless, the first experiment conducted with normal and chimeric faces has led to a similar visual exploration of both types of faces, at least for the first saccade directed toward them.

Finally, a third argument in favor of a gaze bias more linked to perceptual and oculomotor constraints is provided by the total absence of relationship between perceptual responses from the participants to the gender judgment task and their pattern of visual exploration. Indeed, in the first as well as in the second experiment, the GB found for each position of presentation and each saccadic condition was strictly identical regardless of the perceptual responses taken on the basis of the left or the right side of the face. In other words, perceptual responses were not determined by the visual exploration of the face. Alternatively, the gaze exploration was not guided by preferences in favor of one or the other side of the face. Few studies have attempted to determine the existence of a link between perceptual and gaze bias. Their results were not consistent as one study has found a left PB without any GB on the first saccade or total gaze duration [41] whereas the other found a left GB without left PB [17]. Only Butler and collaborators [14], [42], [43] found a subtle link between both biases by separating analyses on GB relative to the perceptual responses of participants in a gender judgment task. Although they did not find any differences on the first saccade that was always directed to the left side of the face, they found that people with left PB did explore more frequently and for longer periods of time the left side, not the right side, of the face. No differences were found for people with right PB. In our experiments, we failed to find such a subtle relationship between perceptual and gaze biases. This may be due to the failure to find a clear left perceptual bias in our experiments. Indeed, before examining the relationship between perceptual and gaze biases, it would have been useful to replicate the two effects. However, we did not find a clear perceptual bias as well as a clear gaze bias in all of our experimental conditions. Despite these potential limitations of our results, we would argue that even in the conditions in which a left perceptual bias significantly appeared (i.e. top position in the saccade condition of the first experiment, central position in the two-saccade condition and right position in the fixation, one-saccade and two-saccade conditions in the second experiment), we never found a precise relationship between the left average PB and the average GB. Alternatively, even in conditions in which we found a clear average gaze bias, this was not associated with a clear perceptual bias. For example, in the first experiment, we found an important average left gaze bias for right face's position as well as an important average right gaze bias for left face's position. If the perceptual and gaze biases were closely linked, then we should also find an important left perceptual bias for right position and an important right perceptual bias for left position. This was not the case as the PB was close to 0 for left and right face's positions. Moreover, additional analyses did not support the existence of any link such as the analyses of the average gaze bias relative to the average perceptual bias that did not reveal correlations between both indexes as well as the analysis of the gaze behavior by separating for each participant the perceptual responses biased to the left or to the right. Beyond their potentials limitations, our data showed the role of individual variability. Here, we would like to emphasize that the failure to find the subtle relationship between perceptual and gaze biases as reported by Butler and collaborators [14] may be linked to the distribution of individual biases. Indeed, Butler and collaborators [14] found a relationship only for participants presenting a left PB whereas in our experiments, the proportion of participants with left PB was not significantly larger than the number of participants showing a right PB and no bias. Another explanation may be that the relationship might require more than three saccades (and thus a longer exposure duration time) to emerge. Indeed, their results were obtained with a presentation time of 2 seconds, involving probably more than three saccades. However, Phillips & David [17] failed to find a left PB for 5 seconds of presentation. They interpreted this result as the disappearance of an initial left perceptual bias, which may have been lost while exploring the face.

However, we believe that our overall results are consistent with previous studies failing to find clear perceptual bias or gaze bias as well as any link between both of them [17], [19], [41]. In line with previous studies, we would like to emphasize here the importance of taking into account both experimental constraints of the used procedure (e.g. initial position of presentation, [18], [19]) and the individual profile of participants ([45], [46]) in terms of sign and magnitude of perceptual and gaze biases before interpreting any possible relationship between these biases in terms of hemispheric activation. Further studies should examine whether perceptual individual profiles are related to individual profiles of cerebral activation as demonstrated previously by Yovel et al. [38] for the fusiform area.

To conclude, the perceptual bias is dependent on the procedure that is used, as it is enhanced with exposure time (the number of allowed saccadic movements here) and faces position. Inter-individual differences are noted, some observers showing a left perceptual bias, others a right one and for some – none at all. The gaze bias seems to be driven by stimulus properties effect (center of gravity effect) and the exploration of a face is affected by the initial position of the face and thus the orientation of the first saccade toward it. Finally, no link between both biases was found, as the visual exploration of a face does not change according to the side of the face from which observers base their judgment.
